# Effects of phenyl acids on different degradation phases during thermophilic anaerobic digestion

**DOI:** 10.3389/fmicb.2023.1087043

**Published:** 2023-04-05

**Authors:** Eva Maria Prem, Alessa Schwarzenberger, Rudolf Markt, Andreas Otto Wagner

**Affiliations:** Department of Microbiology, Universität Innsbruck, Innsbruck, Austria

**Keywords:** anaerobic digestion, high-throughput amplicon sequencing, phenyl acids, thermophilic, extracellular polymer substances, excitation-emission matrices, microbial diversity

## Abstract

Aromatic compounds like phenyl acids (PA) can accumulate during anaerobic digestion (AD) of organic wastes due to an increased entry of lignocellulose, secondary plant metabolites or proteins, and thermodynamic challenges in degrading the benzene ring. The effects of aromatic compounds can be various – from being highly toxic to be stimulating for methanogenesis – depending on many parameters like inoculum or molecular characteristics of the aromatic compound. To contribute to a better understanding of the consequences of PA exposure during AD, the aim was to evaluate the effects of 10 mM PA on microbial communities degrading different, degradation phase–specific substrates in thermophilic batch reactors within 28  days: Microcrystalline cellulose (MCC, promoting hydrolytic to methanogenic microorganisms), butyrate or propionate (promoting syntrophic volatile fatty acid (VFA) oxidisers to methanogens), or acetate (promoting syntrophic acetate oxidisers to methanogens). Methane production, VFA concentrations and pH were evaluated, and microbial communities and extracellular polymeric substances (EPS) were assessed. The toxicity of PA depended on the type of substrate which in turn determined the (i) microbial diversity and composition and (ii) EPS quantity and quality. Compared with the respective controls, methane production in MCC reactors was less impaired by PA than in butyrate, propionate and acetate reactors which showed reductions in methane production of up to 93%. In contrast to the controls, acetate concentrations were high in all PA reactors at the end of incubation thus acetate was a bottle-neck intermediate in those reactors. Considerable differences in EPS quantity and quality could be found among substrates but not among PA variants of each substrate. *Methanosarcina* spp. was the dominant methanogen in VFA reactors without PA exposure and was inhibited when PA were present. VFA oxidisers and *Methanothermobacter* spp. were abundant in VFA assays with PA exposure as well as in all MCC reactors. As MCC assays showed higher methane yields, a higher microbial diversity and a higher EPS quantity and quality than VFA reactors when exposed to PA, we conclude that EPS in MCC reactors might have been beneficial for absorbing/neutralising phenyl acids and keeping (more susceptible) microorganisms shielded in granules or biofilms.

## 1. Introduction

According to the Climate Target Plan of the European Union (EU), a greenhouse gas reduction of 55% is necessary by 2030 to become climate-neutral by 2050. In this regard, biogas has come into focus as regional, sustainable and carbon-neutral energy source to reduce fossil-fuel combustion thus net carbon release into the atmosphere. Biogas production out of organic waste materials coming from municipalities (Angelidaki et al., 2006), agricultural facilities (O’Connor et al., 2020), wastewater treatment plants (Silvestre et al., 2015), slaughterhouses (Cuetos et al., 2010) or food industries (Pazera et al., 2015) is of special interest as their use does not influence food prices, prevents the expansion of monocultures and supports the infrastructure in remote areas. On this basis, various pre-treatment techniques aim at increasing the bioavailability of recalcitrant organic (waste) material for biogas production (Wagner et al., 2018; Khan and Ahring, 2019).

One obstacle of using (pre-treated) organic wastes for biogas production is the increased entry of potentially inhibitory molecules like ammonia (Angelidaki and Ahring, 1993; Bender and Conrad, 1994; Müller et al., 2016; Zilio et al., 2020) or aromatic compounds (Sierra-Alvarez and Lettinga, 1991; Hernandez and Edyvean, 2008; Hecht and Griehl, 2009; Prem et al., 2021). Accumulations of aromatic compounds are exceptionally difficult to manage as the cleavage of benzene rings is thermodynamically challenging under anaerobic conditions (e.g., Kuntze et al., 2008; Carmona et al., 2009; Fuchs et al., 2011; Kuntze et al., 2011; Appel et al., 2021). The most relevant sources of aromatic compounds in organic wastes are lignin, the second most abundant polymer in nature after cellulose (Glasser, 2019) and also found in non-woody plants in form of lignocellulose (Howard et al., 2003), secondary plant metabolites like flavonoids (Fuchs et al., 2011; Salas et al., 2011; Wikandari et al., 2015) and aromatic amino acids found in animal or plant proteins (Iannotti et al., 1986; Hecht and Griehl, 2009; Wagner et al., 2019b). All aromatic compounds contain at least one benzene ring which can create weak intermolecular N-H ∙∙∙ π or O-H ∙∙∙ π bonds (Perutz, 1993; Ottiger et al., 2009). Due to their hydrophobic character, aromatic compounds can pass the cell membrane and cause intracellular dysfunctions, DNA mutations and physiological restrictions (Macgregor and Jurd, 1978; Sierra-Alvarez and Lettinga, 1991; Biharee et al., 2020). Despite these characteristics, the effects of aromatic compounds on AD microorganisms are not yet fully understood insofar as different substrates, inocula, loading rates, reactor types, feeding modi etc. were used in previous investigations. Hecht and Griehl, for instance, showed that phenylacetic acid (PAA) is an important intermediate during AD of kitchen waste and early indicator for process impairments. 0.73 mM PAA had stimulating effects on microorganisms coming from a swine manure treating biogas plant during AD of kitchen waste but led to reduced biogas production when the sludge derived from a reactor solely fermenting kitchen waste (Hecht and Griehl, 2009). Cabrol et al. (2015) showed negative effects of PAA pulses on the archaeal community structure and methanogenesis. However, in both studies, no direct effects of PAA could be found on methanogens (Hecht and Griehl, 2009; Cabrol et al., 2015). In meso- and thermophilic batch reactors, the AD of aromatic substrates led to the formation of PAA, phenylpropionic acid (PPA) and phenylbutyric acid (PBA) and had negative effects on methanogenesis and – in part – also on acetate degradation. Syntrophic acetate oxidation coupled to hydrogenotrophic methanogenesis played an important role and replaced acetoclastic methanogenesis in most cases (Prem et al., 2020, 2021). Contrarily, humic acids (8 g L^−1^) inhibited hydrolytic bacteria and hydrogenotrophic methanogens during mesophilic AD of cellulose and xylan but did not affect acetoclastic methanogens; however, humic acid composition varied, and the authors concluded that effects might be situation-dependent (Azman et al., 2017).

Microorganisms can prevent or recover from unfavorable conditions *via* many different strategies like rapid adaptions of the lipid composition of the cell membrane (Siliakus et al., 2017). Another approach is the formation of biofilms, flocs and granules which have also become interesting for biotechnological purposes in general and under anaerobic conditions in particular (Park and Novak, 2009; Edel et al., 2019; Trego et al., 2020). Under certain conditions, microorganisms can excrete extracellular polymer substances (EPS) which facilitate the formation of such cell structures (Flemming, 2016). EPS mainly comprise of proteins, polysaccharides, and humic and nucleic acids (Ma et al., 2019). Biofilms and flocs can function as physical barrier and protect microorganisms from inhibitory substances or dehydration (Henriques and Love, 2007; Edel et al., 2019; Hu et al., 2019), promote extracellular electron transfer (EET) (Xiao et al., 2017) for e.g., anode-respiring bacteria (Rittmann, 2018), function as nutrient storage (Zhang and Bishop, 2003) and natural retentostat, reduce the recalcitrance of substrates (Chávez et al., 2008; Rittmann, 2018) and enable highly structured zones and nutrient gradients thus niches within the biofilm (Flemming, 2016; Edel et al., 2019). Previous studies could show that inhibitory substances such as poly aluminum chloride (Chen et al., 2018), perchlorate (Liu et al., 2019) or urea formaldehyde resin (Yang et al., 2022) can promote aggregate and EPS formation during AD. Benzenes could be absorbed by EPS in soils, sediments and ground water (Huang et al., 2022). To our best knowledge, EPS formation and their role during AD of aromatic compounds has not been thoroughly studied so far. Regarding the attempts to promote biogas production (from lignin- or protein-rich waste materials) and to overcome its obstacles, further research on EPS formation is therefore inevitable. However, the quality and quantity of EPS also strongly depends on the involved microorganisms, available substrates as well as on various process parameters (Edel et al., 2019) which exacerbate a comprehensive understanding of their characteristics and importance for biotechnological purposes like biogas production. In regard to the different conclusions on effects of aromatic compounds on AD microorganisms, a closer look on the role of EPS is pending in general and might be an additional puzzle piece in understanding the microbial dynamics during AD of aromatic compounds.

Consequently, to better understand the impact of phenyl acid during (thermophilic) AD, the aims of the present study were to (i) promote microorganisms of certain degradation phases, from hydrolysis to methanogenesis by feeding degradation phase- specific substrates (ranging from complex cellulose to acetate), (ii) assess the effects of phenyl acids (PA) on the respective microbial community, and iii) evaluate the overall community structure *via* 16S rRNA amplicon sequencing and the EPS quality *via* excitation-emission matrices (EEM) for each substrate and PA variant.

## 2. Materials and methods

### 2.1. Batch reactor setup and experimental design

**Figure 1:**
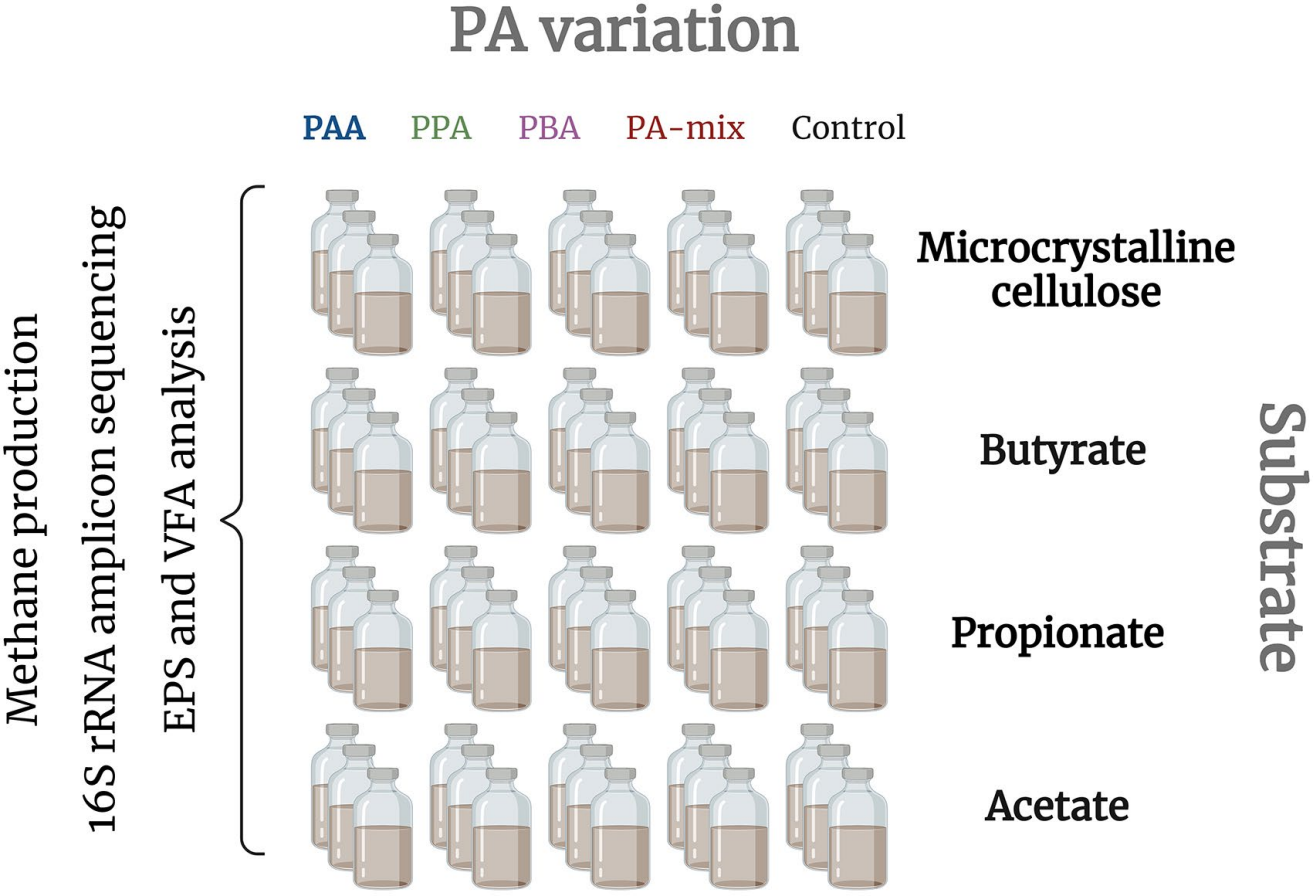
Batch reactors were set up with different substrates to promote specific AD phases: all stages (MCC), heterotrophic acetate production to methanogenesis (butyrate and propionate) and (SAO- coupled and/or acetoclastic) methanogenesis (acetate). For each substrate, five different PA variants were included in three parallels: Reactors were exposed to either 10 mM PAA, PPA, PBA, PA-mix or 0 mM phenyl acids (controls). Biochemical analyzes (methane production, pH, EPS) as well as amplicon sequencing were done with all reactors.

The inoculum (press water sludge) originated from a thermophilic, organic fraction of the municipal solid waste (OFMSW) reactor (Wagner et al., 2014). Back at the laboratory, the sludge was immediately incubated at 55°C for 1 month to remove degradable residuals. 120 ml batch reactors (Figure 1) were filled with 75 ml sludge (1:8 diluted) and with different substrates depending on the targeted AD stages and microorganisms to be promoted: Microcrystalline cellulose (hydrolysis - methanogenesis), butyrate and propionate (syntrophic VFA oxidisers - methanogenesis) and acetate (syntrophic acetate oxidation (SAO) and/or (acetoclastic) methanogenesis) were added to the reactors in concentrations that equal a chemical oxygen demand of 0.4 [gO_2_] for each substrate and reactor according to the *Buswell-Boyle* equation (Buswell and Mueller, 1952; Achinas and Euverink, 2016). Additionally, phenyl acids were added in five different variations: (i) controls (no phenyl acid addition), (ii) 10 mM phenylacetic acid (PAA), (iii) 10 mM 3-phenylpropionic acid (PPA), (iv) 10 mM 3-phenylbutyric acid (PBA) or (v) 10 mM PAA-PPA-PBA mix (1:1:1, thereon called “PA-mix”). All variations were analyzed in triplicates; hence 60 batch reactors were used in total (Figure 1). Prior incubation, reactors were flushed with N_2_ and immediately sealed with butyl-rubber stoppers and aluminum caps. The reactors were incubated at 55°C for 28 days. Gas analyzes (CH_4_, CO_2_ and H_2_) took place at 8 time points (on day 0, 3, 5, 7, 10, 14, 21 and 28). Liquid samples for VFA, pH and metagenomic analyzes were taken on day 0, 14, 21 and 28. EPS analyzes *via* excitation-emission matrices were done with samples of day 21. 16S rRNA amplicon sequencing were conducted for day 0 (represented by butyrate control samples) and 28 (all samples).

### 2.2. General biochemical analyzes

A GC-TCD (Shimadzu, Japan) was used for CH_4_, CO_2_ and H_2_ analyzes (Wagner et al., 2012). Gas-overpressure was assessed *via* a GHM Greisinger GDH 200 sensor and used to calculate biogas and methane production (NmL) as described previously (Wagner et al., 2019a). Concentrations of volatile fatty acids (VFA: acetate, propionate and butyrate) and of phenyl acids (PAA, PPA and PBA) were analyzed *via* HPLC-UV/VIS (Shimadzu, Japan) at 220 and 270 nm (Wagner et al., 2017, 2019b). The pH was assessed with pH indicator strips pH 5.0–10.0 (pH resolution: 0.5, Merck, Germany).

### 2.3. Excitation/emission matrix spectroscopy of EPS

EPS extraction based on previously established protocols (Guo et al., 2014; Sun J. et al., 2016) with following modifications: first centrifugation step was done at 20000 g, the second step at 12000 g, and the third and fourth step at 5000 g. Heat treatment was done with a Bioer Mixing Block MB-102 dry bath at a speed of 300 rpm for 15 min. Thereafter, the samples were centrifuged again at 8000 g for 10 min; each supernatant was transferred into a fresh tube and stored at −20°C. Fluorescence-based EEM analyzes were done with a Hitachi F-4500 fluorescence spectrophotometer at 25°C. A 3D-wavelength scan was conducted with excitation wavelengths ranging from 200 to 400 nm in 10 nm steps and emission wavelengths from 200 to 600 nm in 5 nm steps. According to Sun J. et al. (2016), specific excitation-emission wavelength profiles can be attributed to certain areas within the EEM matrix: Class I includes tyrosine-like proteins (EX 200–250 nm/EM 200–330 nm), Class II tryptophan-like proteins (EX 200–250 nm/EM 330–380), class III fulvic acid-like organics (EX 200–250 nm/EM 380–500 nm), class IV soluble, microbial by-products (EX 250–280 nm/EM 200–380 nm) and class V includes humic acid- like organics (EX 250–400 nm/EM 380–500 nm).

### 2.4. DNA extraction

After the removal of the liquid phase for VFA and phenyl acid analyses, each sample pellet was resuspended in 600 μl phosphate buffer (Prem et al., 2019) and transferred into bead tubes. After another centrifugation step at 11,000 g for 10 min, the supernatants were removed. DNA was extracted with the NucleoSpin® Soil DNA extraction kit (Macherey-Nagel, Germany). After adding 700 μl of lysis buffer SL-1 and 50 μl enhancer, cell lysis took place with a FastPrep®-24 (RRID:SCR_018599, MP Biomedicals) at 5 m/s for 30 s. Consequent steps were done according to the manufacturer’s protocol with 50–100 μl of elution buffer. DNA quantity and quality were checked with a NanoDrop™ 2000c (RRID:SCR_020309, Thermo Scientific™) spectrophotometer.

### 2.5. dPCR analyses

For absolute quantifications of prokaryotic reads, digital PCR (dPCR) was performed with a QIAcuity One 5plex System (Qiagen: RRID:SCR_008539, Netherlands), QIAcuity 8.5 k 96-well Nanoplate, QIAcuity EvaGreen PCR Kit (QIAGEN, Germany) and Qiacuity Software Suite. Cycling conditions and PCR mix preparation with the primer pair 515f / 806r targeting the V4 SSU rRNA gene (Apprill et al., 2015) were according to the manufacturer’s protocols.

### 2.6. Library preparation and high-throughput amplicon sequencing

The library was prepared *in-house*. Both PCR steps were performed according to previous protocols (Prem et al., 2019) with following modifications: 25 μl PCR mix contained 12.5 μl NEBNext® High-Fidelity 2x PCR Master Mix (New England Biolabs, United States), 1.25 μl forward (515-f), 1.25 μl reverse primer (806-r), 9 μl PCR-free water and 1 μl template (5 ng sample^−1^). Thermocycling conditions were as recommended by the manufacturer with 30 cycles (2^nd^ PCR: 7 cycles) of denaturation at 98°C for 10 s, annealing at 57°C for 25 s and elongation for 30 s. PCR mix aliquoting, template addition, primer addition (2^nd^ PCR step) and sample pooling was done with a Biomek 4,000 Automation Workstation (RRID:SCR_019618, Beckman Coulter, United States). Size selection was performed with a ProNex® Size-Selective Purification System (Promega, United States). PCR clean-up was performed with the Monarch PCR & DNA Clean-up Kit (NEB, United States). The final sample pool contained 15 ng μL^−1^, with an 260/280 absorbance ratio of 1.62. The sample was subsequently sent to Microsynth AG (Switzerland). After another clean-up step at the facility, sequencing was done with a MiSeq™ device (RRID:SCR_016379, Illumina®, United States). The ZymoBIOMICS™ Microbial Community DNA Standard II Log Distribution (Zymo Research, United States) was included for checking the PCR procedure, sequencing and reads procession.

### 2.7. Database preparation and *in-silico* reads procession

The SILVA (RRID:SCR_006423) V138.1 database was downloaded from the SILVA database homepage^1^ and further processed with UBUNTU 20.04.2 LTS as operating system. The database was loaded into ARB (RRID:SCR_000515) version arb-6.0.6 (Westram et al., 2011) for removal of low-quality reads and chimeras. In mothur (RRID:SCR_011947) v.1.45.1 (Schloss et al., 2009), the database was cut to position 11 k to 26 k according to the *Escherichia coli* alignment coordinates in SILVA 138.1. Filtering of unique sequences without regard to their alignment and removal of redundant sequences were done in ARB and mothur v.1.45.1, respectively.

The sequencing library contained samples of several studies (in total: 157 samples). However, after sequencing, the samples of each study were analyzed separately. In this case, 63 batch reactor samples and 2 Mock samples were metagenomically analyzed. Reads procession was done according to earlier protocols (Prem et al., 2019) with following changes: Mothur version 1.46.1 (Schloss et al., 2009) was used for all following metagenomic analyzes if not indicated otherwise. 1,926,976 sequences remained after the quality check. After rarefaction analyses, samples were normalized to 7,879 reads per sample. The OTU tables prior and after subsampling positively correlated (*R* = 0.89, *p* < 0.01, *Mantel*-test).

### 2.8. Statistics and graphical presentation

**Figure 2:**
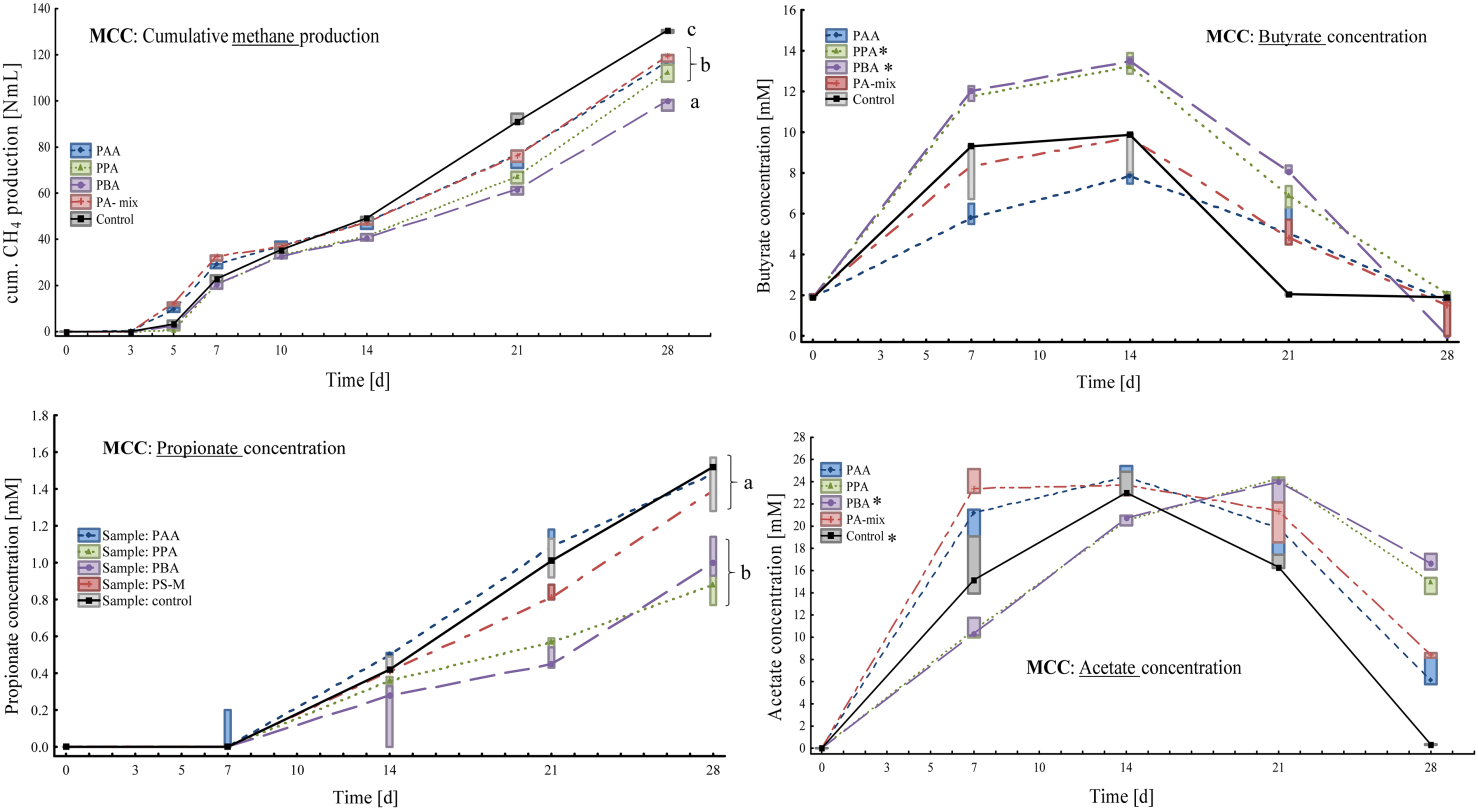
Box-plots showing cumulative methane production (top, left), butyrate (top, right), propionate (bottom, left) and acetate concentrations (bottom, right) of MCC reactors exposed to different phenyl acids during 28 days of incubation. Boxes show median and 25–75% confidence intervals. Lowercase letters (ANOVA, *Bonferroni* post-hoc test) and stars (*Kruskal-Wallis* ANOVA, multiple comparisons) indicate significant differences among PA variants on day 28.

**Figure 3:**
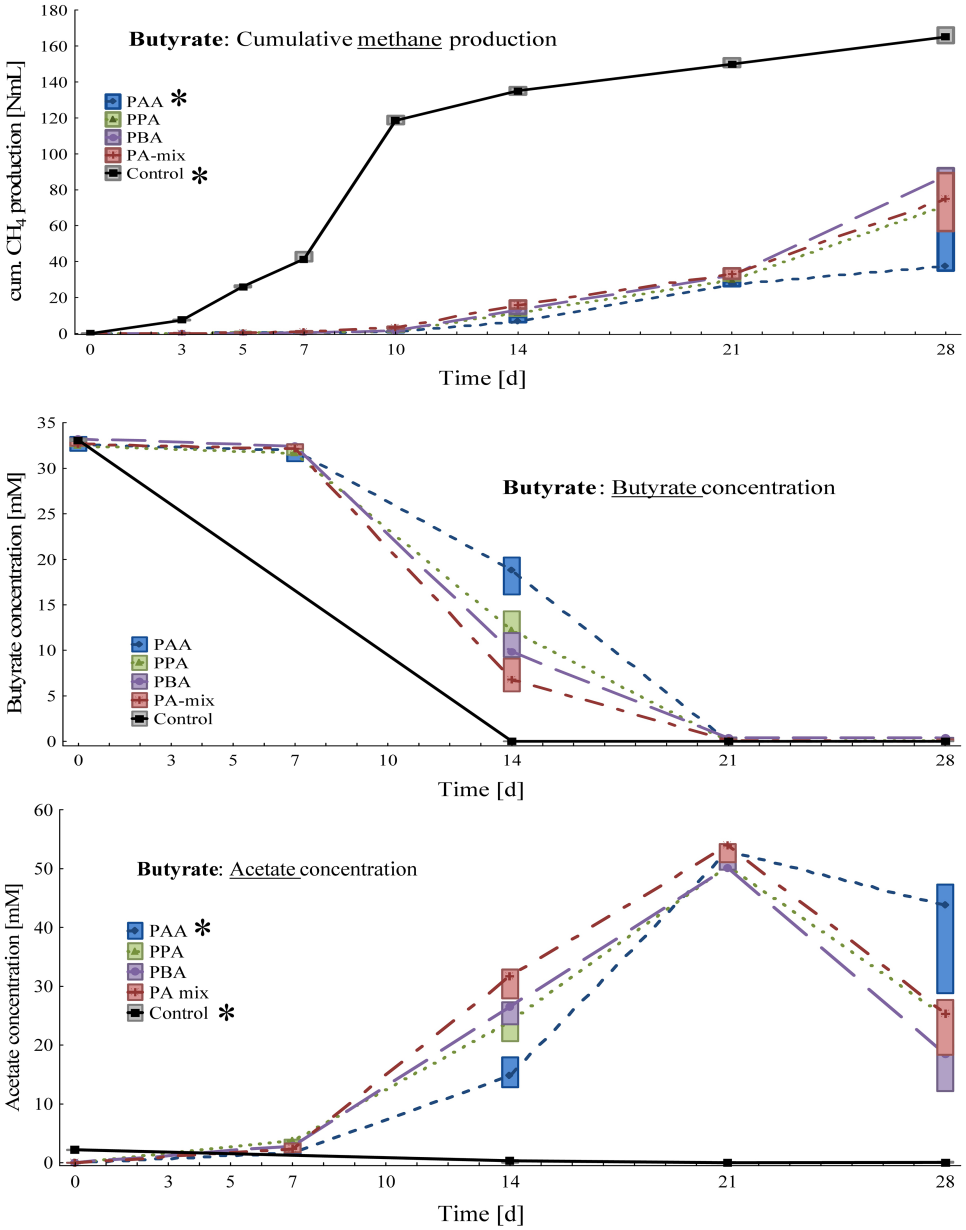
Box-plots showing cumulative methane production (top), butyrate (middle) and acetate (bottom) concentrations of butyrate reactors exposed to different phenyl acids during 28 days of incubation. Boxes show median and 25–75% confidence intervals. Stars indicate significant differences (*Kruskal-Wallis* ANOVA, multiple comparisons) among PA variants on day 28.

**Figure 4:**
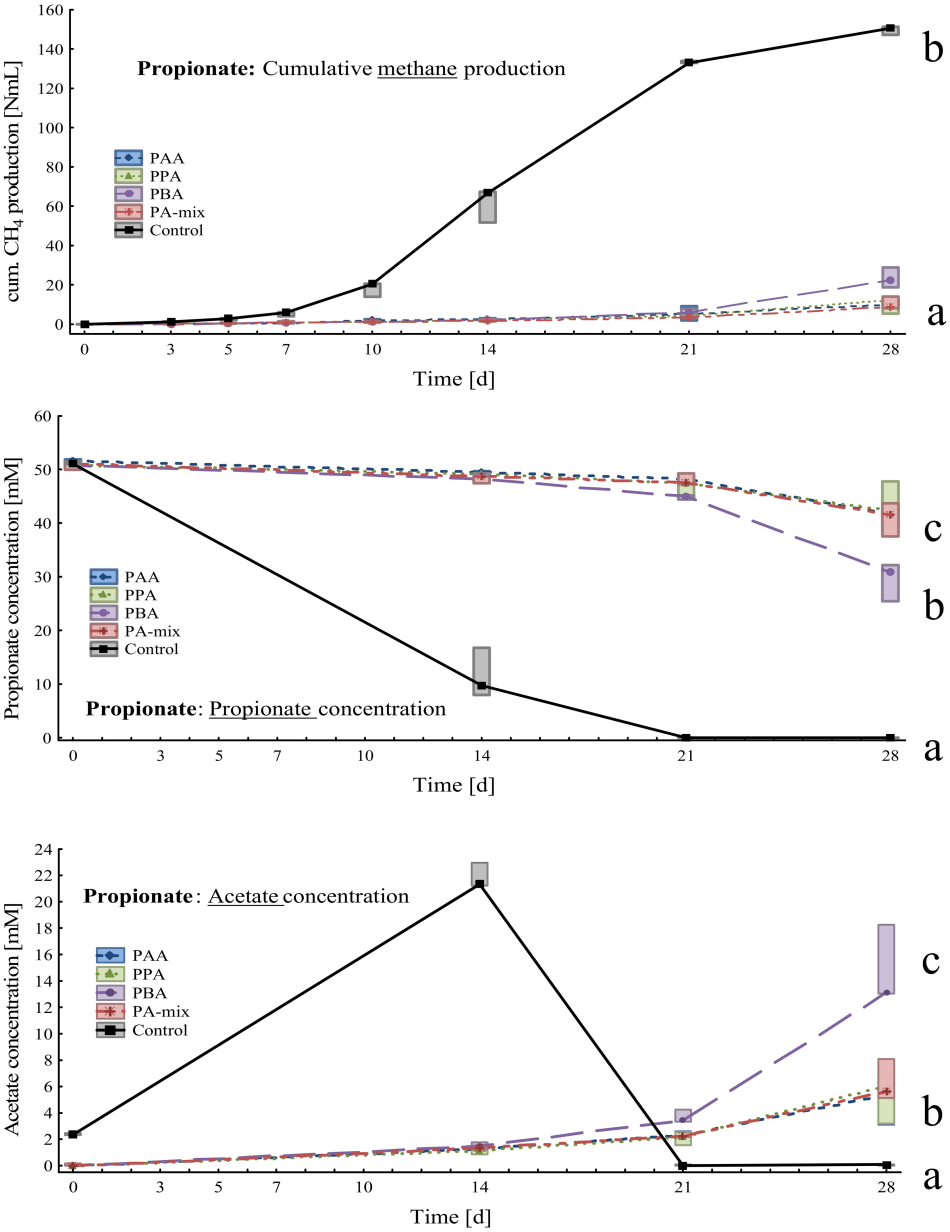
Box-plots showing cumulative methane production (top), propionate (middle) and acetate (bottom) concentrations of propionate reactors exposed to different phenyl acids during 28 days of incubation. Boxes show median and 25–75% confidence intervals. Lowercase letters indicate significant differences (ANOVA, *Bonferroni* post-hoc test) among PA variants on day 28.

**Figure 5:**
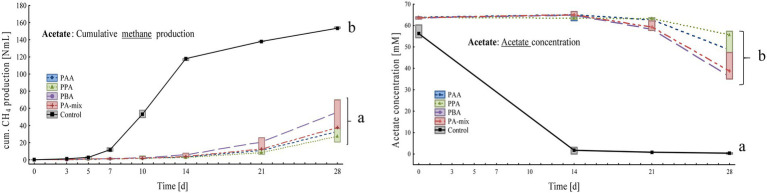
Box-plots showing cumulative methane production (left) and acetate concentrations (right) of acetate reactors exposed to different phenyl acids during 28 days of incubation. Boxes show median and 25–75% confidence intervals. Lowercase letters indicate significant differences (ANOVA, *Bonferroni* post-hoc test) among PA variants on day 28.

The significance cut-off was set at *α* = 0.05. Data in the text show mean + SD if not indicated otherwise. For statistical analyses, methane, VFA, pH and EEM data were log_10_(x + 1) and sequencing results were box-cox(x + 1) transformed, if not indicated otherwise. After checking homogeneity of variance and normal distribution of residuals for each analysis, one-way ANOVA and the *Bonferroni* post-hoc test were used for statistical analyzes on day 28. If the requirements for parametric analyzes were not met, *Kruskal-Wallis* ANOVA with multiple comparisons of mean ranks for all groups was applied. Statistical analyzes of biochemical data (Figures 2–5) were done in Statistica™ 13 (RRID:SCR_014213, StatSoft, Inc., United States). PAST (RRID:SCR_019129) version 4.09 (Hammer et al., 2001) was used for the *Mantel* test. The core microbiome and *LEfSe* biomarkers (class: substrate, subclass: phenyl acid variant) were determined *via* mothur 1.46.1 (Schloss et al., 2009). Radar charts for *LEfSe* biomarkers were done in SigmaPlot™ 14 (RRID:SCR_003210, Systat® Software Inc.). Heat trees (main text and Supplementary material) were done in RStudio© (RRID:SCR_000432) 2022.02.3 + 492 with R version 4.2.1 using the *Wilcoxon* test and *Benjamini*-*Hochberg* correction with following packages: *metacoder* (Foster et al., 2017), *phyloseq* (McMurdie and Holmes, 2013), *extrafont* (Chang, 2014) and *ggplot2* (Wickham, 2009). Diversity analyzes (*Shannon*-*Weaver* index) were done as described before (Prem et al., 2021). Redundancy analyzes (RDA) ordination with OTUs and EEM data were also done in R with *vegan* (Oksanen et al., 2014), *phyloseq*, *dplyr* (Wickham et al., 2015) and *extrafont*. OTU and EEM data were *Hellinger* transformed (Legendre and Gallagher, 2001) prior RDA ordination took place with the *Bray-Curtis* distance. The heatmap, showing the most abundant taxa (>1,000 reads) over all samples, was done in R version 4.2.2 with the packages *readxl* (Wickham and Bryan, 2019), *pheatmap* (Kolde, 2019) and *extrafont*. Hierarchical clustering was used for defining clusters.

## 3. Results

A comprehensive list of H_2_, CO_2_, VFA and PA concentrations as well as pH values of all assays can be looked up in Supplementary Tables S1–S4.

### 3.1. General metagenomic results

**Figure 6:**
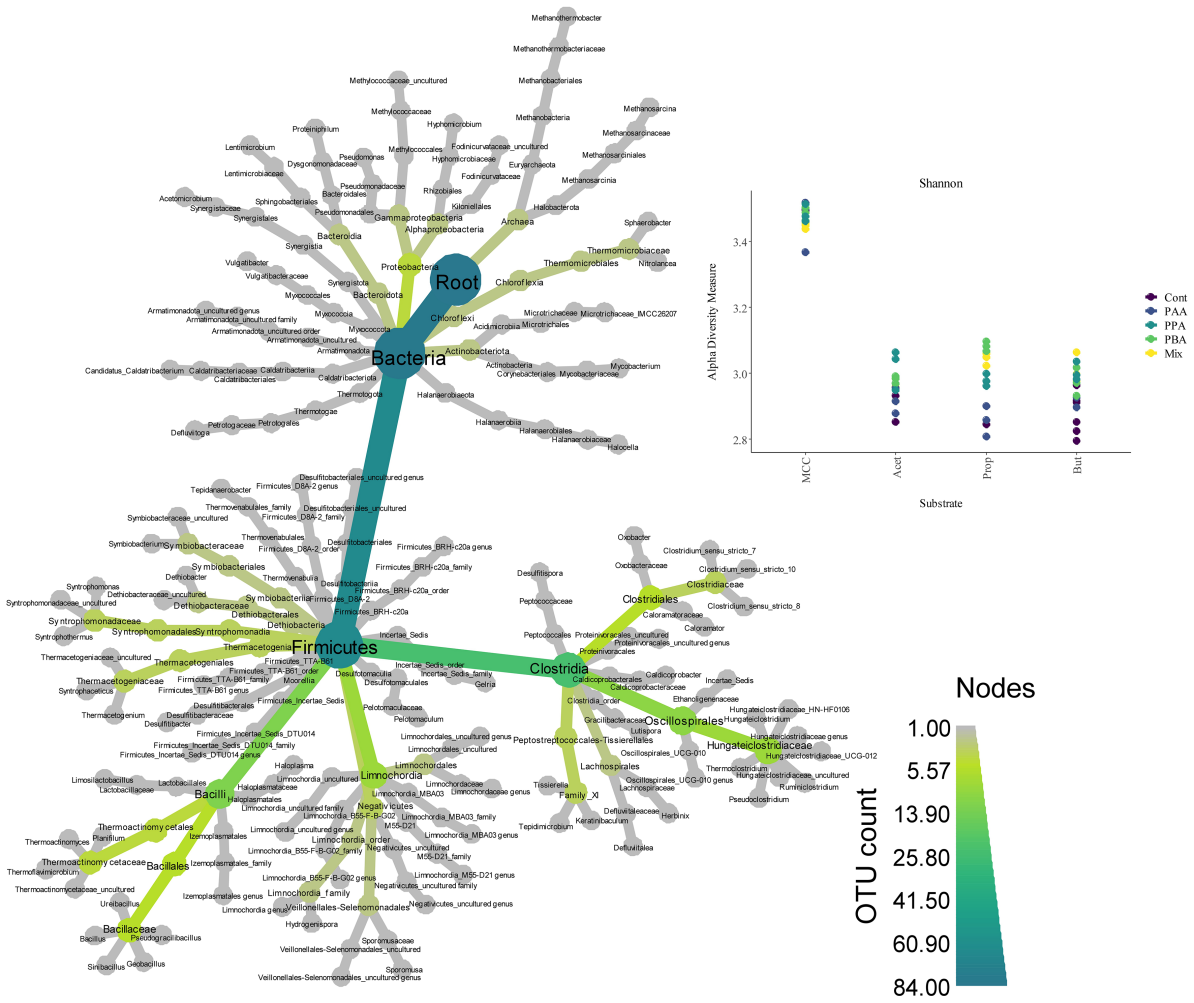
Heat tree (left) showing microbial diversity and abundance (OTU count) over all samples. Shannon diversity indices (top, right) for MCC, acetate (Acet), propionate (Prop) and butyrate (But) reactors. Colors indicate the respective PA variation–from controls (“Cont,” violet), to PA-mix (“Mix,” yellow).

**Figure 7:**
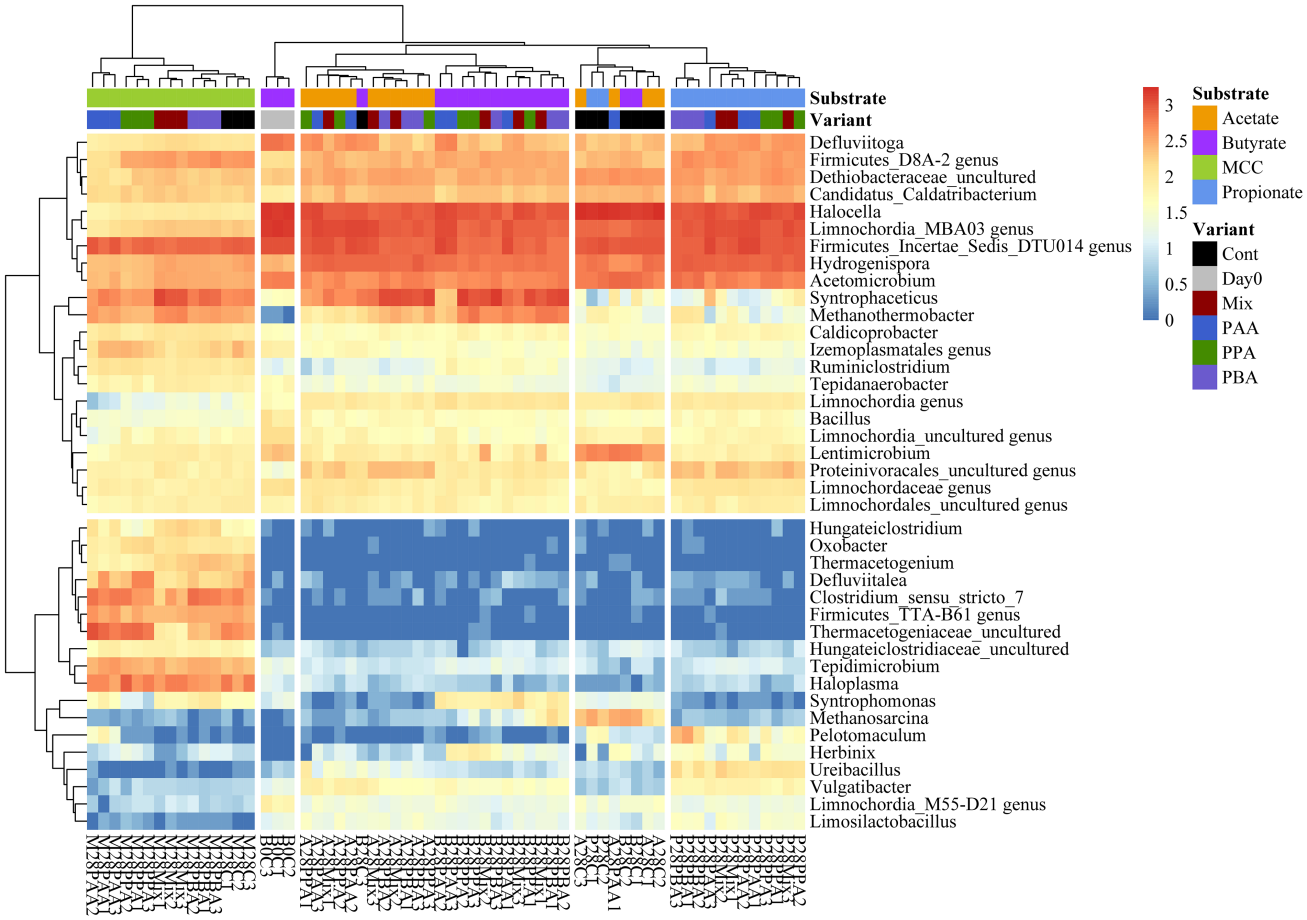
Heatmap of sequence abundance. Microbial taxa with an abundance >1,000 sequences per genus were depicted. Read count was log_10_(x + 1) transformed.

**Figure 8:**
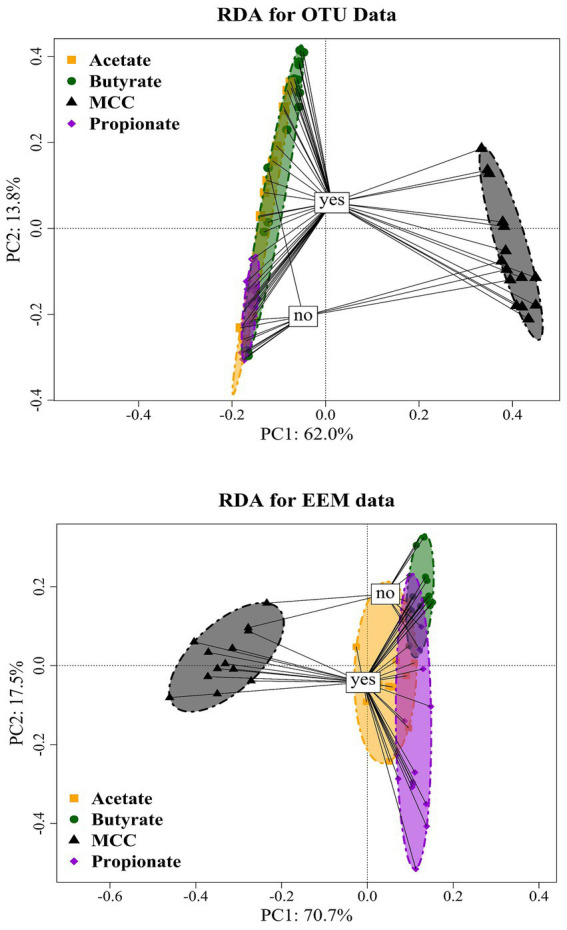
RDA ordination plot of OTUs (top) of MCC, butyrate, propionate and acetate reactors with (“yes”) or without PA addition (“no”). RDA ordination plot of EEM (bottom) of MCC, butyrate, propionate and acetate reactors with (“yes”) or without PA addition (“no”).

Over all batch reactors on day 28, *Firmicutes* and *Clostridia* were the dominant phylum and class, respectively (Figure 6; Supplementary Figure S2), and the genera *Hydrogenispora*, *Firmicutes incertae sedis* DTU014, *Limnochordia* MBA03, *Dethiobacteraceae* uncultured, *Acetomicrobium*, *Firmicutes* D8A_2 and Candidatus *Caldatribacterium* represented the core microbiome. Generally, microbial diversity (*Shannon-H*) was highest in MCC reactors – irrespective of the phenyl acid variant (Figure 6). The higher diversity in MCC reactors is also apparent in Figure 7; therefore, their microbial communities clustered apart from VFA reactors (Figure 8). Within VFA assays, propionate reactors clustered apart from butyrate and acetate reactors (Figure 7). The two prevalent methanogens were *Methanothermobacter* spp. and *Methanosarcina* spp., whereby the latter was the dominant methanogen in VFA control reactors and in one acetate reactor exposed to PAA (Figure 7). In all other reactors of day 28, *Methanothermobacter* spp. was the prevalent methanogen. Within the VFA assays, *Lentimicrobium* spp. was abundant in controls and in one acetate PAA reactor but not in other VFA assays exposed to PA (Figure 7).

### 3.2. Substrate microcrystalline cellulose

At the end of the incubation (day 28), methane production was highest in controls (130 ± 0.64 Nml CH_4_) followed by PA-mix (119 ± 1.57 Nml CH_4_), PAA (115 ± 4.77 Nml CH_4_), PPA (112 ± 3.72 Nml CH_4_) and PBA reactors (98.7 ± 2.54 Nml CH_4_) as shown in Figure 2. An increase of acetate, propionate and butyrate concentration could be observed in all assays, whereby kinetic differences were apparent, and acetate was only used up in controls at the end of incubation (Figure 2). On day 28, the highest acetate concentrations were observed in PBA reactors (16.7 ± 0.72 mM). Propionate concentrations increased steadily but moderately in all variants, with the highest concentrations in PAA, PA-mix and control assays (Figure 2). Butyrate dynamics were similar to acetate results, but concentrations were low in all variants on day 28 (Figure 2). All reactors showed a pH around neutral, ranging from pH 6.50 to 8.50, throughout the incubation period (Supplementary Table S1). On day 28, the pH was 7.25 in PA variants and 7.50 in controls.

**Figure 9:**
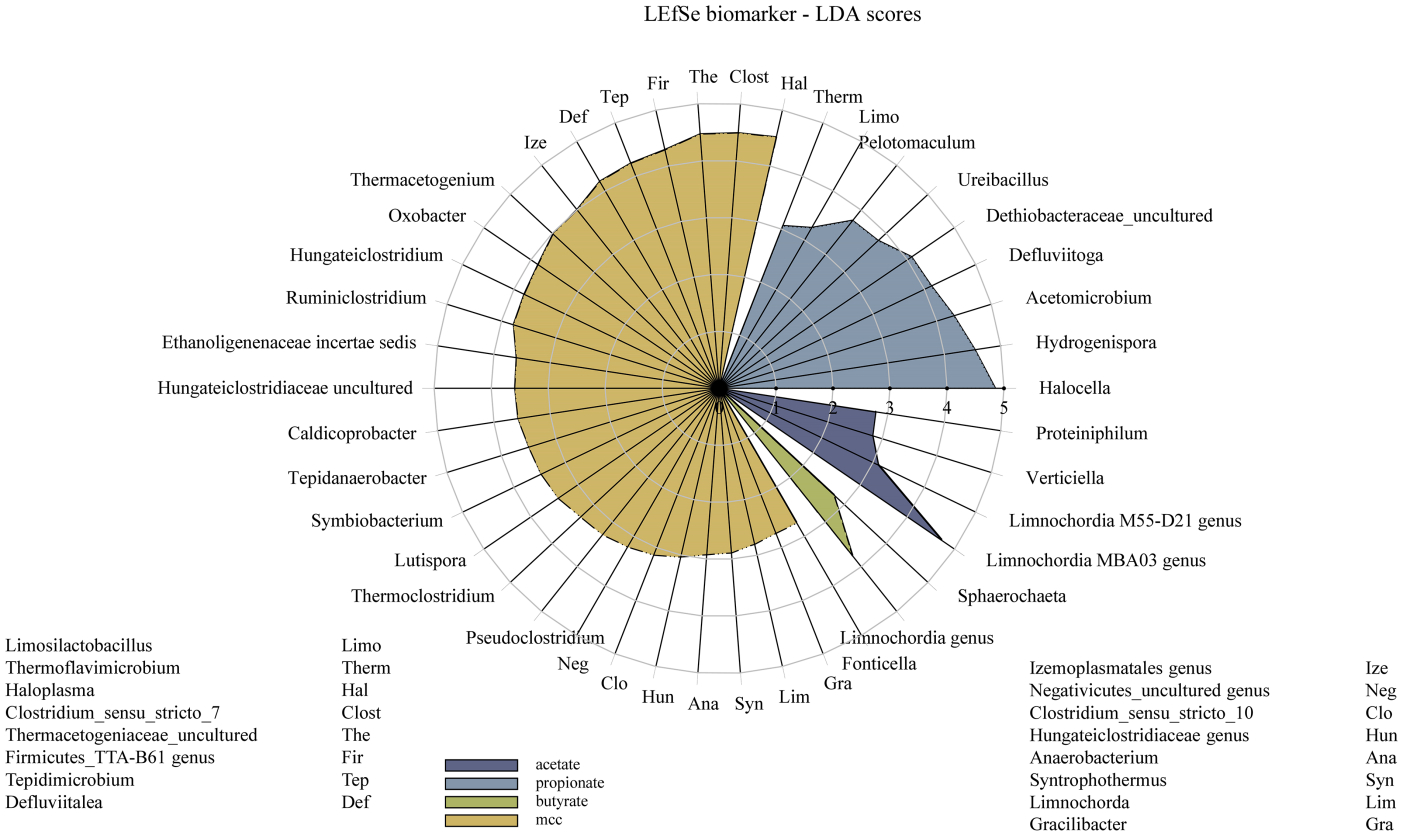
Radar chart showing all *LEfSe* biomarkers and their linear discriminant analysis (LDA) scores over all PA variants of each substrate variation – from acetate (violet) to MCC (yellow).

Additional core microorganisms for MCC reactors were *Syntrophaceticus* spp., *Methanothermobacter* spp., *Ruminiclostridium* spp., *Izemoplasmatales* genus, *Tepidimicrobium* spp., *Haloplasma* spp., *Firmicutes TTA*-B61 genus, *Clostridium sensu strict* 7 genus and *Defluviitalea* ssp. No significant differences in genus proportions could be observed between controls (no PA addition) and PA reactors on day 28 (Figures 7, 8; Supplementary Figure S3). Absolute sequencing reads mL^−1^ batch reactor sludge ranged from 1.32 ∙ 10^7^ ± 2.81 ∙ 10^6^ (Controls on day 0) to 1.40 ∙ 10^8^ ± 1.22 ∙ 10^8^ (PAA reactors on day 28); differences among variants were not significant. *LEfSe* biomarkers for MCC reactors (LDA score ≥ 4) were *Haloplasma* spp., *Clostridium sensu stricto* 7 genus, *Thermacetogeniaceae* uncultured genus, *Firmicutes* TTA-B61 genus, *Tepidimicrobium* spp., *Defluviitalea* spp., *Izemoplasmatales* genus and *Thermacetogenium* spp. (Figures 7, 9). All biomarkers for MCC reactors can be looked up in Figure 9. In all variants of day 28, *Methanothermobacter* spp. was the dominant methanogenic group and *Syntrophaceticus* spp. was one of the most dominant taxa (Figure 7; Supplementary Figure S3).

### 3.3. Substrate butyrate

Cumulative methane production on day 28 (Figure 3) was the highest in controls (166 ± 4.33 Nml CH_4_), followed by PBA (85.5 ± 7.84 Nml CH_4_), PA-mix (73.7 ± 16.2 Nml CH_4_), PPA (72.1 ± 11.7 Nml CH_4_) and PAA reactors (46.1 ± 17.0 Nml CH_4_). The differences between controls and PAA assays were significant. Butyrate was completely degraded in control as well as PA reactors on 28, whereby butyrate degradation was time-delayed in PA reactors as seen on day 14 (Figure 3). While acetate concentrations were very low in control reactors throughout the incubation, acetate accumulated in all PA reactors from day 7 to 21 – with the highest concentrations (53.1 ± 1.73 mM) in PAA variants on day 21. The differences between controls and PAA reactors were significant. Propionate concentrations were low (< 0.2 mM) in all variants throughout the experiment (Supplementary Table S2). The pH values of all reactors were slightly alkalic, ranging from pH 8.00 to 8.25 on day 28 (Supplementary Table S2).

While *Methanosarcina* spp. was the dominant methanogen in most control reactors, *Methanothermobacter* spp. was the prevailing methanogenic genus in PA reactors on day 28 (Figure 7; Supplementary Figure S5). *Syntrophaceticus* spp., *Firmicutes* DTU014 genus, *Limnochordia* MBA03 genus were one of the most abundant genera (Supplementary Figure S5). Absolute sequencing reads mL^−1^ batch reactor sludge ranged from 9.37 ∙ 10^4^ (PA-mix variant) to 6.34 ∙ 10^8^ (PBA variant) on day 28. *Limnochordia* genus was the only *LEfSe* biomarker with an LDA score > 3 for reactors fed with butyrate (Figure 9). Within the acetate-butyrate cluster (Figure 7), the abundance of *Syntrophomonas* spp. and *Herbinix* spp., for instance, was more abundant in butyrate than acetate PA assays.

### 3.4. Substrate propionate

Compared with the controls, methane production in PA reactors was delayed by 2 weeks and slowly started on day 21. On day 28, cumulative methane production was highest in controls (150 ± 2.14 Nml CH_4_), significantly lower amounts were measured in PBA (23.3 ± 5.22 Nml CH_4_), PA-mix (10.4 ± 3.24 Nml CH_4_), PPA (10.3 ± 4.17 Nml CH_4_) and PAA reactors (9.89 ± 3.76 Nml CH_4_) as shown in Figure 4. Propionate was completely degraded in controls on day 28, whereas propionate concentrations were significantly higher in PA reactors; the lowest propionate concentration – after MCC – was observed in PBA reactors with 29.5 ± 3.55 mM (Figure 4). Acetate accumulated in controls (21.8 ± 0.97 mM) until day 14 and was completely degraded on day 28. In PA reactors, acetate accumulation was observed on day 21 and 28, with PBA reactors showing significantly higher acetate concentrations (14.8 ± 2.99 mM) than other PA variants on day 28 (Figure 4). All reactors showed slightly alkalic conditions, ranging from pH 7.50 to 9.00, throughout the incubation period (Supplementary Table S3).

Propionate reactors had the same additional core microbiome as reactors fed with butyrate: *Halocella* spp., *Defluviitoga* spp. and *Limnochordia* spp. Moreover, *Halocella* spp., *Hydrogenispora* spp., *Acetomicrobium* spp., *Defluviitoga* spp. and *Dethiobacteraceae* uncultured genus were biomarkers (LDA score ≥ 4) for propionate reactors (Figure 9). Absolute sequencing reads mL^−1^ batch reactor sludge ranged from 2.48 ∙ 10^5^ (PAA variant) to 5.39 ∙ 10^8^ (PA-mix variant) on day 28. Compared with MCC, butyrate and acetate assays, the abundance of *Syntrophaceticus* spp., *Methanothermobacter* spp. was low in all propionate reactors (Figure 7; Supplementary Figure S7). Genera like *Pelotomaculum* or *Ureibacillus* were more abundant in propionate PA than in control and other assays (Figure 7) and were also significant biomarkers (LDA <4) of propionate reactors (Figure 9).

### 3.5. Substrate acetate

Controls significantly produced more methane than PA assays: Within 28 days, controls produced 154 ± 1.00 Nml, PBA 56.8 ± 3.14 Nml, PA-mix 48.0 ± 18.8 Nml, PPA 39.4 ± 26.8 Nml and PAA reactors 34.7 ± 6.37 Nml CH_4_ (Figure 5). The substrate acetate was low in controls from day 14 on (0.31 ± 0.28 mM), whereas acetate concentrations were significantly higher in PA reactors, ranging from 36.1 ± 1.04 mM in PBA to 49.3 ± 12.4 mM in PPA reactors on day 28 (Figure 5). Propionate and butyrate formations were not observed. All reactors showed slightly alkalic conditions, ranging from pH 8.00 to 9.00, throughout the incubation period (Supplementary Table S4).

*Hallocella* spp. and *Defluviitoga* spp. were additional core microorganism for acetate reactors. Absolute sequencing reads mL^−1^ batch reactor sludge ranged from 1.39 ∙ 10^6^ (PPA variant) to 4.50 ∙ 10^8^ (controls) on day 28. The only *LEfSe* biomarker with an LDA score ≥ 4 was *Limnochordia* MBA03 genus for acetate reactors, followed by *Limnochordia* M55-D21 genus (LDA score > 3) (Figure 9). Like butyrate reactors, *Methanosarcina* spp. was the most abundant methanogen in controls, whereas the relative abundances of *Methanothermobacter* spp. together with potential syntrophic partners like *Syntrophaceticus* spp. was higher in PA reactors on day 28 (Figure 7; Supplementary Figure S9). An uncultured genus of *Proteinivoracales* was more abundant in acetate (controls as well as PA) reactors than in other assays (Figure 7).

### 3.6. Metagenomic differences among substrate variants (MCC, butyrate, propionate, acetate) when exposed to PAA, PPA and PBA

When specifically looking at differences among substrates when exposed to PA-mix (including PAA, PPA and PBA), the class *Clostridia*, especially the orders *Oscillospirales* (family *Hungateiclostridiaceae*), *Clostridiales* (*Clostridium sensu stricto* 7 in particular), as well as the genera *Caldicoprobacter*, *Haloplasma*, *Izemoplasmatales* genus, *Thermacetogenium*, *Thermacetogeniaceae* uncultured genus, *Defluviitalea*, *Lutispora*, *Tepidanaerobacter*, *Tepidimicrobium*, *Symbiobacterium*, *Firmicutes* TTA-B61 genus and *Negativicutes* uncultured genus were significant for MCC as substrate, whereas classes like *Dethiobacteria*, *Limnochordia, Firmicutes* D8A-2 or *Halanaerobiaeota* were significant when VFAs (acetate, propionate or butyrate) were the main substrates. For a complete depiction of microbial diversity and the significance of genera at different PA variations, please refer to Figures 6, 7; Supplementary Figures S1, S2.

### 3.7. Excitation-emission matrices spectroscopy of EPS in MCC, butyrate, propionate and acetate assays

**Figure 10:**
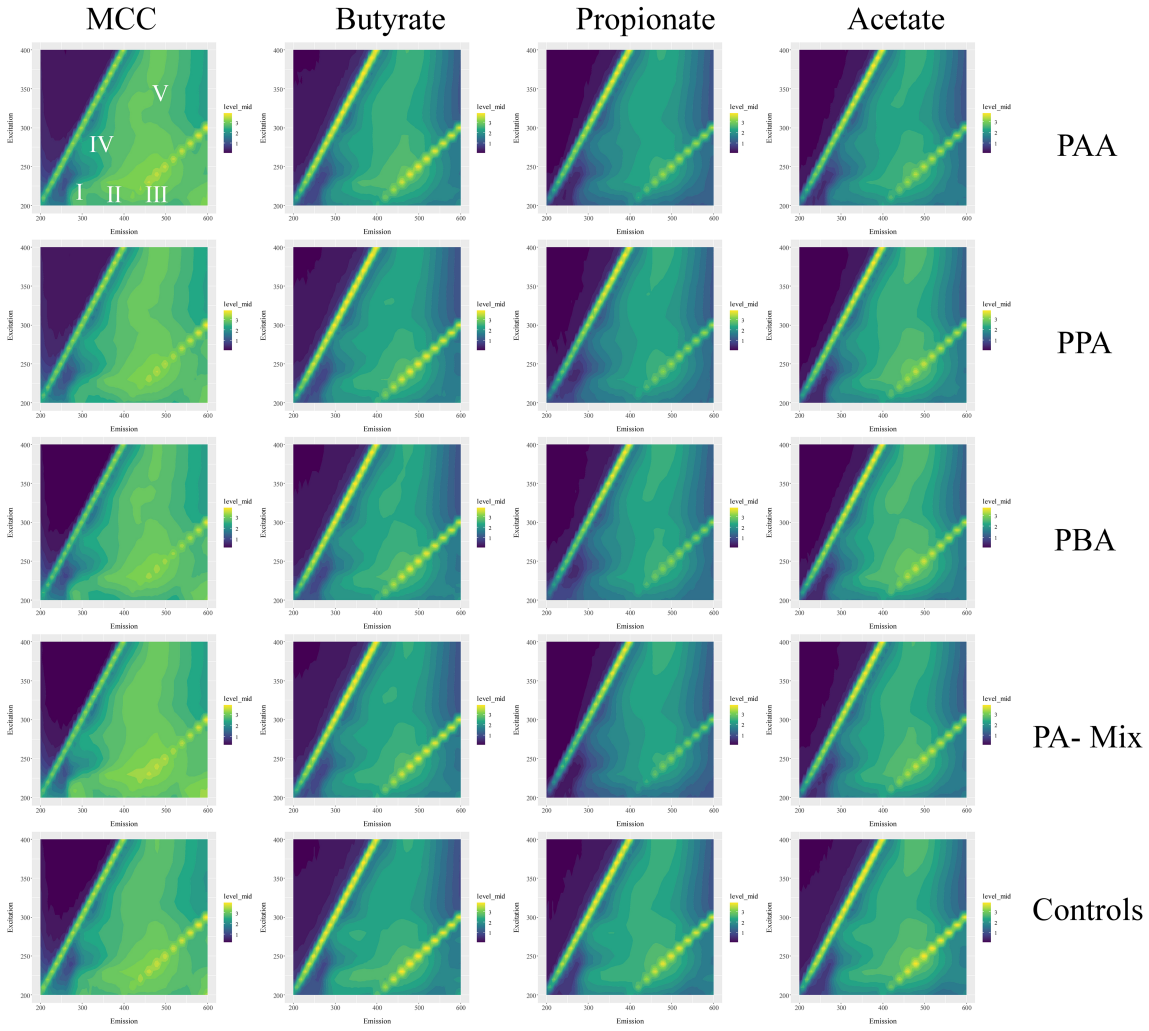
Excitation-emission matrices of MCC, butyrate, propionate and acetate reactors (columns) with the respective PA variations (PAA, PPA, PBA, PA-mix, controls; rows). Average fluorescence data were log_10_(x + 1) transformed and range from 0 (dark violet) to 3 (yellow). I–V show areas typical for specific EPS types–this caption counts for all matrices.

Quantitative and qualitative differences were detected among variants: Generally, MCC reactors showed higher absolute fluorescence values than acetate, propionate and butyrate reactors (Figure 10; Supplementary Figures S4, S6, S8, S10); this was especially the case for propionate assays (Figure 10; Supplementary Figure S8). Class I (tyrosin-like proteins) and class IV (soluble microbial by-products) EPS tended to be more dominant in MCC reactors than in VFA assays. Furthermore, the fluorescence spectrum (Ex 200 to 250 nm / EM 500–600 nm) also showed higher fluorescence values (Figure 10). RDA ordination confirmed these differences as MCC reactors clustered apart from VFA assays, whereby the choice of substrate (MCC or VFA) explained more of the differences in EEM than PA exposure (yes / no, Figure 8).

## 4. Discussion

PA exposure let to severe losses in methane production at the end of the incubation when VFAs (acetate, propionate and butyrate) were digested. In acetate reactors, phenyl acid exposure (10 mM) let to a loss in methane production of 63% (PBA), 69% (PA-mix), 74% (PPA) and 77% (PAA) compared with the controls (Figure 5). In propionate and butyrate reactors, methane production was reduced by 84% (PBA) and 93% (PAA, PPA, PA-mix, Figure 4), and 48% (PBA), 56% (PA-mix), 57% (PPA) and 72% (PAA, Figure 3) compared with the controls, respectively. In MCC reactors, the loss in methane production was less drastic compared with the controls: 9% (PA-mix), 12% (PAA), 14% (PPA) and 24% (PBA) as shown in Figure 2. VFA data were in accordance with the methane production results: In controls, the respective VFA substrate was used up at the end of the incubation, whereas the exposure to PA led to a significantly delayed degradation of the respective substrate, especially in propionate and acetate reactors. However, the microbial community seemed to adapt to the PA exposure in those reactors and methane production started time-delayed (Figures 4, 5). This is further supported by the fact that PA were not degraded (Supplementary Tables S1–S4) within 28 days thus microorganisms were not exposed to lower concentrations of PA at the end of incubation. The incubation ended on day 28 but further studies on the adaptability of microorganisms to PA exposure are of interest. In contrast to propionate and acetate reactors, butyrate was fully degraded in all variants from day 21 on; however, acetate concentrations accumulated until day 21 and were still high on day 28 (Figure 3). Propionate degradation started very late (between day 21 and 28) in PA reactors, and acetate started to accumulate (Figure 4). During syntrophic butyrate and propionate oxidation, acetate is an important intermediate and its concentrations should remain very low due to thermodynamic reasons (Hao et al., 2020). In AD systems, acetoclastic methanogens and/or SAO–bacteria (with a hydrogenotrophic partner) are potential syntrophic partners and are important for a rapid acetate turnover (Stams and Plugge, 2009; Worm et al., 2014; Hao et al., 2020). Acetate degradation in acetate reactors was also inhibited for the first 14 days when PA were present, and concentrations slowly started to decrease (Figure 5). Therefore, the biochemical results strongly indicate that VFA and especially propionate and acetate turnover were delayed / inhibited in VFA reactors when exposed to 10 mM PA.

The chosen PA concentration of 10 mM was considered suitable as earlier studies showed AD impairments at 3.6 mM PAA (Sabra et al., 2015), 5 mM PAA (Ziels et al., 2018), or 6.5 mM p-cresol (Rétfalvi et al., 2013). Moreover, 0.7 mM PAA had stimulating as well as inhibitory effects during AD depending on the origin of the inoculum (Hecht and Griehl, 2009). The effects of 5 mM PAA depended on the substrate (primary/secondary sludge of a wastewater treatment plant) in another study (Cabrol et al., 2015). The here presented findings confirm that the substrate fed to (thermophilic) communities is crucial when assessing the susceptibility to aromatic compounds. Regarding the influence of the inoculum, further studies with other inocula would be informative. A concentration of 10 mM is also practice-oriented: For example, AD of phenylalanine led to PAA concentrations ranging from 0 to 6 to 23 mM (Wagner et al., 2019b). About 1.8 mM PAA (together with other aromatic compounds) were formed during AD of kitchen waste (Hecht and Griehl, 2009), 1.2 mM p-cresol and 0.4 mM PPA during AD of corn straw (Qiao et al., 2013), and 12 mM PBA during AD of wheat straw (Prem et al., 2019).

The microbial diversity was significantly higher in MCC than in VFA fed reactors (Figure 6). This is plausible as hydrolytic and fermentative bacteria are needed for the degradation of polymers (e.g., MCC). A variety of intermediates are produced at this stage which in turn support the growth of other microorganisms (Figure 7). Many organisms significant for MCC reactors (with and without PA addition, Figures 7, 9) are indeed engaged in hydrolytic and acidogenic processes: For example, *Haloplasma* spp. (*H. contractile*) is a nitrate/nitrite reducing and lactate producing bacterium with unique morphological properties (Antunes et al., 2008). Like *Haloplasma* (*contractile*), *Izemoplasmatales* genus has no peptidoglycan cell wall (Wang et al., 2020) and belongs to the class *Bacilli* (Figure 9) according to the *Genome Taxonomy Database* (Parks et al., 2022). *Tepidimicrobium* spp. is a hydrolytic and acidogenic bacterium, which was an important genus during AD of lignocellulose (Prem et al., 2019) and aromatic amino acids (Prem et al., 2020). *Defluviitalea* spp. is also a thermophilic anaerobe and produces butyrate or acetate during fermentation (Jabari et al., 2012; Ma et al., 2017). Further potential acetate producers were e.g., *Caldicoprobacter* spp. (Bouanane-Darenfed et al., 2015) or *Lutispora* spp. (Shiratori et al., 2008). SAO-coupled hydrogenotrophic methanogenesis was the major methanogenic pathway in all MCC reactors as abundances of (potential) SAO-bacteria like *Syntrophaceticus* spp. (Westerholm et al., 2010), *Thermacetogenium* spp. (Mosbæk et al., 2016; Dyksma et al., 2020), *Thermacetogeniaceae* uncultured genus, *Tepidanaerobacter* spp. (Westerholm et al., 2011) or *Firmicutes incertae sedis* DTU014 genus, (Perman et al., 2022), and of the hydrogenotrophic methanogen *Methanothermobacter* spp. (Kaster et al., 2011) were high in those reactors (Figure 7).

PA addition in VFA reactors not only led to a decreased methane production but also to a change in methanogenic pathway: *Methanosarcina* spp. was the dominant methanogenic genus in most controls (Figure 7) probably due to high VFA concentrations (Figures 3–5) and the high activity of the acetate kinase (ACK)/phosphate acetyltransferase (PTA) complex of *Methanosarcina* sp. (Berger et al., 2012). However, when exposed to PA, *Methanosarcina* spp. was clearly inhibited in acetate and propionate PA reactors and to a lesser extent also in butyrate PA reactors (Figure 7). Typical VFA oxidisers in butyrate reactors were *Syntrophomonas* spp. (butyrate oxidiser, Zhang et al., 2004, 2005) and *Syntrophaceticus* spp. (also abundant in acetate reactors) which probably cooperated with *Methanothermobacter* spp. In propionate reactors, the late start of methane production (Figure 4) was also reflected in a lower abundance of *Methanothermobacter* spp. (Figure 7). Moreover, biochemical as well as sequencing data showed that PBA had a lesser effect on syntrophic propionate oxidation (e.g., *Pelotomaculum* spp., *Methanothermobacter* spp.) than other PA variants, and acetate accumulations were highest in these reactors (Figure 4). *Pelotomaculum* spp. was also quite abundant in PAA reactors of the MCC assay (Figure 7). Previous studies showed that SAO- coupled hydrogenotrophic methanogenesis is a competitive pathway at thermophilic temperatures and at moderate acetate concentrations (Dolfing, 2014) and is more resistant to potential inhibitors like ammonia (e.g., Müller et al., 2016) or (moderate concentrations of) phenyl acids (e.g., Prem et al., 2021, 2023).

Generally, microbial diversity was higher in VFA reactors when PA were present (Figure 6) probably because the community was stressed and otherwise very active microorganisms could not dominate at these unfavorable conditions – a trend which was also seen in previous studies on AD of aromatic compounds (e.g., Prem et al., 2020). However, biochemical as well as sequencing data did not allow general conclusions about the differences in toxicity among PA variants (PAA, PPA, PBA, PA-mix) (Figures 2–5, 7). Like *Methanosarcina* spp., *Lentimicrobium* spp. was abundant in VFA control reactors but not in VFA assays exposed to PA (Figure 7). This genus belongs to the phylum *Bacteroidetes*. Interestingly, the only described species is *L. saccharophilum* which is mesophile and cannot degrade acetate, propionate and butyrate (Sun L. et al., 2016). Another susceptible organism to PA was *Pseudomonas* spp. which was significant for VFA reactors without PA addition (Supplementary Figure S1). In previous investigations, *Pseudomonas* spp. was one of the most abundant bacteria during thermophilic AD of organic waste (Kabaivanova et al., 2022), a key-player for a stable AD of carbohydrates (Buettner et al., 2019) and able to exploit VFAs (Jie et al., 2014).

The inclusion of hydrolytic and fermentative bacteria during AD of MCC might explain the higher quantity (higher fluorescence intensity) and broader spectrum of EPS compared with VFA reactors (Figure 10). Classes like *Clostridia*, *Bacilli*, *Thermacetogenia* or *Negativicutes* (Figures 6, 7) where relevant in MCC reactors; however, it is not clear which taxa were important for biofilm/granule formation and whether hydrolytic and acidogenic bacteria directly needed the protective barrier themselves or needed EPS for other purposes like nutrient storage (Zhang and Bishop, 2003). The type of substrate (MCC - VFA) had more influence on EPS formation than PA addition - this was also the case for the microbial community analyzes (Figure 8). EPS mainly contain polymers like polysaccharides, proteins and humic acids, and enclose cells in a matrix by electrostatic and hydrophobic interactions (Henriques and Love, 2007). EPS contain charged functional side groups and thus enable ligation to other charged molecules (Li et al., 2022). PAA (pKa = 4.31), PPA (pKa = 4.66) and PBA (pKa = 4.67) are mainly dissociated in water at neutral to slightly alkalic pH (Supplementary Tables S1–S4); therefore, phenyl acids could have been effectively bound to EPS. As the effects of PA were less severe in MCC reactors (Figure 2), we hypothesize that phenyl acids were more effectively absorbed/neutralized by EPS in MCC than in VFA reactors. It is also possible that EPS better facilitated granule and biofilm formation thus more susceptible microorganisms were physically protected from inhibitory compounds. In both cases, further studies are essential. Furthermore, syntrophic VFA oxidation coupled hydrogenotrophic methanogenesis is an important pathway at elevated PA concentrations during AD, as shown above and in previous investigations (e.g., Prem et al., 2021). This process is thermodynamically challenging thus many VFA oxidisers depend on syntrophic partners in near proximity to maintain direct interspecies electron or H_2_/formate transfer for feasible reactions (e.g., McInerney et al., 2009; Walker et al., 2020; Westerholm et al., 2021). This further supports that EPS are essential not only to neutralize inhibitory substances like PA but also to keep microorganism in near proximity in form of highly structural granules (Trego et al., 2020) or biofilms (Hu et al., 2019), and to facilitate the transition of electrons (Xiao et al., 2017). Our results on biochemical and molecular biological data combined with data on EPS quality and quantity are preliminary but pose interesting questions for further research - not only on the effects of aromatic compounds on AD communities but also on the overall structure and interdependence of involved microorganisms.

In conclusion, we could show that the toxicity of PA during AD depended on the type of substrate (MCC, butyrate, propionate and acetate) which in turn determined the (i) microbial diversity and composition and (ii) EPS quantity and quality. In VFA assays, where the substrate spectrum was narrower than in MCC reactors, PA led to drastic losses in methane production and to acetate accumulations compared to the controls. In MCC reactors, the effects of PA were not as severe as in VFA assays probable due to a higher microbial diversity and higher EPS quantity. Syntrophic VFA oxidation coupled hydrogenotrophic methanogenesis was the dominant methanogenic process in all MCC as well as in VFA reactors exposed to PA. Acetoclastic methanogenesis was only prevalent in VFA assays without PA exposure. This again confirms that acetate is a bottle-neck intermediate during AD especially under unfavorable conditions (Westerholm et al., 2016; Prem et al., 2020, 2021; Singh et al., 2021). As phenyl acids were not degraded in any variant within 28 days, and as EPS quality and quantity was higher in reactors including all degradation phases (substrate MCC), we hypothesize that EPS played an important role in absorbing/neutralizing phenyl acids and in forming biofilms and granules to physically protect more susceptible microorganisms. These are interesting data which could be the basis for further research on (i) why *Methanosarcina* spp. was more susceptible to phenyl acids and (ii) how EPS as protective agents can improve the AD of aromatic compounds and other potentially inhibitory substances.

## Data availability statement

The datasets presented in this study can be found in online repositories. The names of the repository/repositories and accession number(s) can be found in the article/Supplementary material.

## Author contributions

EP, RM, and AW designed the study. AS and AW set up the reactors. AS, EP, and AW conducted sampling and biochemical analyses. EP and RM did EEM spectroscopy and dPCR analyses. EP created and checked the library for amplicon sequencing, did read processing as well as statistical and graphical analyses. AW and EP raised funds. AW supervised the findings of the study. All authors helped shaping the analyzes and manuscript, read the final manuscript and agreed to be accountable for the content of the work.

## Funding

The stand-alone projects *Phenylodigest* (P 29143) and *Phenylomicrobe* (P 33838) of the Austrian Science Fund (FWF) as well as the Tyrolean Science Fund (TWF, *PhenylopH*) financially supported this study.

## Conflict of interest

The authors declare that the research was conducted in the absence of any commercial or financial relationships that could be construed as a potential conflict of interest.

## Publisher’s note

All claims expressed in this article are solely those of the authors and do not necessarily represent those of their affiliated organizations, or those of the publisher, the editors and the reviewers. Any product that may be evaluated in this article, or claim that may be made by its manufacturer, is not guaranteed or endorsed by the publisher.

## Footnotes

^1^ https://arb-silva.de
