# Supplementary material for: Effects of phenyl acids on different degradation phases during thermophilic anaerobic digestion
Source: Front Microbiol. 2023 Apr 5;14:1087043. doi: 10.3389/fmicb.2023.1087043 (PMC10113666; doi:10.3389/fmicb.2023.1087043)
Supplement: Supplementary file 1 [file Data_Sheet_1.pdf]

Supplementary Material

Supplementary Results and Discussion

Supplementary Table 1. H_2 and CO_2 as well as VFA (acetate, propionate and butyrate) and PA (PAA, 3-PPA, 3PBA) concentrations, and pH of MCC samples on day 0, 14, 21 and 28

Time [d]	PA-Variant	H_2 [%]	CO_2 [%]	pH	Acetate [mM]	Propionate [mM]	Butyrate [mM]	PAA [mM]	3-PPA [mM]	3-PBA [mM]
0	PAA	0.00	0.00	7.00	0.00	0.00	2.05	10.5	0.00	0.00
0	PAA	0.00	0.00	7.50	0.00	0.00	1.87	10.4	0.00	0.00
0	PAA	0.00	0.00	7.50	0.00	0.00	1.86	10.3	0.00	0.00
0	PPA	0.00	0.00	7.50	0.00	0.00	1.83	0.00	12.0	0.00
0	PPA	0.00	0.00	8.00	0.00	0.00	1.85	0.00	12.1	0.00
0	PPA	0.00	0.00	8.00	0.00	0.00	1.91	0.00	12.2	0.00
0	PBA	0.00	0.00	7.50	0.00	0.00	1.89	0.00	0.00	9.45
0	PBA	0.00	0.00	7.50	0.00	0.00	1.87	0.00	0.00	9.46
0	PBA	0.00	0.00	7.50	0.00	0.00	1.88	0.00	0.00	9.12
0	PA-mix	0.00	0.00	8.00	0.00	0.00	1.94	3.60	3.54	3.78
0	PA-mix	0.00	0.00	8.00	0.00	0.00	1.93	3.54	3.50	3.73
0	PA-mix	0.00	0.00	8.00	0.00	0.00	1.98	3.60	3.55	3.81
0	Control	0.00	0.00	8.00	0.00	0.00	1.85	0.00	0.00	0.00
0	Control	0.00	0.00	8.00	0.00	0.00	1.87	0.00	0.00	0.00
0	Control	0.00	0.00	8.50	0.00	0.00	1.90	0.00	0.00	0.00
14	PAA	0.04	41.8	6.50	25.4	0.50	8.15	9.79	0.00	0.10
14	PAA	0.03	38.2	6.50	22.9	0.45	7.87	8.92	0.00	0.08
14	PAA	0.00	38.8	7.00	24.5	0.51	7.47	9.32	0.00	0.11
14	PPA	0.00	40.6	6.50	20.6	0.34	13.9	0.09	11.5	0.00
14	PPA	0.00	39.2	6.50	20.5	0.38	13.2	0.08	11.6	0.00
14	PPA	0.00	40.1	6.50	20.6	0.36	12.9	0.08	11.5	0.00
14	PBA	0.00	38.2	6.50	20.8	0.00	13.5	0.07	0.00	9.14
14	PBA	0.00	39.7	6.50	20.1	0.28	13.7	0.07	0.00	9.13
14	PBA	0.00	39.9	7.00	20.9	0.33	13.3	0.07	0.00	9.10
14	PA-mix	0.00	41.6	6.50	23.7	0.41	9.77	3.34	3.34	3.76
14	PA-mix	0.00	42.7	7.00	22.8	0.41	9.74	3.25	3.26	3.65
14	PA-mix	0.00	34.7	6.50	24.2	0.42	9.09	3.33	3.33	3.74
14	Control	0.00	51.2	6.50	24.9	0.49	8.03	0.05	0.00	0.09
14	Control	0.00	37.7	6.50	23.0	0.42	9.87	0.04	0.00	0.10
14	Control	0.00	34.3	6.50	22.8	0.41	9.94	0.04	0.00	0.10
21	PAA	0.00	32.0	7.00	16.7	1.09	7.10	9.74	0.00	0.10
21	PAA	0.00	31.5	7.00	20.0	1.06	5.04	10.1	0.00	0.13
21	PAA	0.00	31.2	7.00	19.8	1.18	4.97	10.1	0.00	0.14
21	PPA	0.00	32.0	6.50	24.3	0.51	7.36	0.11	10.4	0.00
21	PPA	0.00	31.5	6.50	23.3	0.57	6.89	0.11	11.3	0.00
21	PPA	0.00	31.9	6.50	24.4	0.59	6.31	0.11	11.9	0.00
21	PBA	0.03	37.0	6.50	22.1	0.45	8.02	0.08	0.00	9.15
21	PBA	0.00	31.5	6.50	24.0	0.43	8.06	0.10	0.00	9.75
21	PBA	0.00	31.7	6.50	24.1	0.54	8.36	0.10	0.00	9.77
21	PA-mix	0.00	31.7	6.50	22.1	0.81	4.81	3.32	3.36	3.85
21	PA-mix	0.00	31.2	7.00	21.4	0.88	4.49	3.19	3.23	3.65
21	PA-mix	0.00	30.9	6.50	18.5	0.80	5.71	3.32	3.32	3.63
21	Control	0.00	30.9	7.00	16.2	1.13	1.99	0.06	0.00	0.11
21	Control	0.00	30.4	7.00	17.4	1.01	2.10	0.04	0.00	0.11
21	Control	0.00	30.4	6.50	16.3	0.92	2.05	0.05	0.00	0.11
28	PAA	0.00	29.8	7.25	8.29	1.48	1.82	10.1	0.00	0.14
28	PAA	0.00	30.0	7.25	6.12	1.41	1.68	9.66	0.00	0.14
28	PAA	0.00	29.2	7.25	5.77	1.57	1.67	9.80	0.00	0.15
28	PPA	0.00	30.0	7.25	15.0	0.77	2.15	0.10	3.98	18.6
28	PPA	0.00	30.0	7.25	15.4	0.98	2.06	0.10	4.14	19.5
28	PPA	0.00	29.6	7.25	13.9	0.88	2.06	0.10	3.46	21.9
28	PBA	0.03	31.1	7.25	17.5	0.93	0.00	0.09	0.00	9.98
28	PBA	0.00	31.0	7.25	16.1	1.00	0.00	0.11	0.00	10.0
28	PBA	0.00	31.7	7.25	16.6	1.14	0.00	0.10	0.00	9.86
28	PA-mix	0.00	29.0	7.25	8.44	1.39	0.00	3.24	0.00	13.2
28	PA-mix	0.00	29.3	7.25	8.57	1.34	1.50	3.11	0.49	11.9
28	PA-mix	0.00	29.1	7.25	8.16	1.56	1.67	3.29	0.00	13.4
28	Control	0.00	27.0	7.50	0.31	1.28	1.89	0.06	0.00	0.12
28	Control	0.00	28.2	7.50	0.33	1.57	1.90	0.05	0.00	0.12
28	Control	0.00	29.4	7.50	0.36	1.52	1.98	0.05	0.00	0.12

Supplementary Table 2. H_2 and CO_2 as well as VFA (acetate, propionate and butyrate) and PA (PAA, 3-PPA, 3PBA) concentrations, and pH of butyrate-fed samples on day 0, 14, 21 and 28

Time [d]	PA-Variant	H_2 [%]	CO_2 [%]	pH	Acetate [mM]	Propionate [mM]	Butyrate [mM]	PAA [mM]	3-PPA [mM]	3-PBA [mM]
0	PAA	0.00	0.00	9.00	0.00	0.00	32.7	10.1	0.00	0.00
0	PAA	0.00	0.00	8.50	0.00	0.00	31.9	9.93	0.00	0.00
0	PAA	0.00	0.00	9.00	0.00	0.00	33.5	10.4	0.00	0.00
0	PPA	0.00	0.00	9.00	0.00	0.00	32.4	0.00	10.6	0.00
0	PPA	0.00	0.00	9.00	0.00	0.00	32.4	0.00	10.7	0.00
0	PPA	0.00	0.00	9.00	0.00	0.00	32.7	0.00	10.8	0.00
0	PBA	0.00	0.00	8.50	0.00	0.00	33.3	0.00	0.00	10.3
0	PBA	0.00	0.00	8.50	0.00	0.00	33.1	0.00	0.00	10.3
0	PBA	0.00	0.00	8.50	0.00	0.00	33.2	0.00	0.00	10.3
0	PA-mix	0.00	0.00	8.50	0.00	0.00	33.0	3.45	3.43	3.65
0	PA-mix	0.00	0.00	9.00	0.00	0.00	32.7	3.46	3.46	3.65
0	PA-mix	0.00	0.00	9.00	0.00	0.00	32.5	3.42	3.42	3.64
0	Control	0.00	0.00	7.50	2.21	0.00	33.0	0.13	0.00	0.00
0	Control	0.00	0.00	7.50	2.12	0.07	33.2	0.13	0.00	0.00
0	Control	0.00	0.00	8.50	2.20	0.00	33.1	0.13	0.00	0.00
14	PAA	0.08	2.25	8.50	17.9	0.00	16.2	10.1	0.00	0.00
14	PAA	0.08	1.89	8.50	14.7	0.00	18.8	9.97	0.00	0.00
14	PAA	0.08	2.06	9.00	12.8	0.00	20.2	9.95	0.00	0.00
14	PPA	0.06	2.24	8.50	25.0	0.00	10.9	0.00	10.5	0.00
14	PPA	0.06	2.40	8.50	23.9	0.00	12.2	0.00	10.7	0.00
14	PPA	0.07	1.93	9.00	20.7	0.00	14.3	0.00	10.9	0.00
14	PBA	0.05	2.23	8.50	27.3	0.00	9.25	0.00	0.00	10.0
14	PBA	0.06	2.12	8.50	26.5	0.00	9.86	0.00	0.00	10.2
14	PBA	0.04	1.99	8.50	23.5	0.00	11.9	0.00	0.00	10.1
14	PA-mix	0.20	2.40	8.50	32.9	0.00	5.50	3.38	3.29	3.45
14	PA-mix	0.05	2.04	8.50	31.6	0.00	6.75	3.47	3.42	3.57
14	PA-mix	0.00	1.94	8.50	28.0	0.00	9.09	3.53	3.50	3.67
14	Control	0.05	21.2	8.00	0.45	0.39	0.00	0.07	0.04	0.00
14	Control	0.00	21.0	8.00	0.00	0.00	0.00	0.08	0.00	0.00
14	Control	0.00	20.6	7.50	0.33	0.16	0.00	0.08	0.00	0.00
21	PAA	0.00	3.87	8.00	51.9	0.00	0.00	9.94	0.00	0.00
21	PAA	0.02	3.54	8.00	53.1	0.00	0.00	10.1	0.00	0.00
21	PAA	0.01	3.35	8.00	53.0	0.00	0.00	10.2	0.00	0.00
21	PPA	0.01	3.70	8.25	50.7	0.00	0.00	0.00	10.2	0.00
21	PPA	0.03	3.63	8.25	50.0	0.00	0.00	0.00	10.4	0.00
21	PPA	0.00	3.28	8.25	50.5	0.00	0.00	0.00	10.3	0.00
21	PBA	0.02	4.35	8.00	50.2	0.00	0.36	0.00	0.00	10.2
21	PBA	0.02	3.99	8.00	49.8	0.00	0.42	0.00	0.00	10.2
21	PBA	0.01	3.55	8.00	52.4	0.00	0.24	0.00	0.00	10.1
21	PA-mix	0.02	3.79	8.25	54.2	0.00	0.11	3.68	3.58	3.70
21	PA-mix	0.02	3.49	8.25	51.1	0.00	0.22	3.59	3.56	3.64
21	PA-mix	0.02	3.44	8.25	54.0	0.00	0.13	3.68	3.64	3.73
21	Control	0.00	19.5	8.00	0.09	0.00	0.00	0.18	0.00	0.00
21	Control	0.00	18.8	8.50	0.00	0.00	0.00	0.19	0.00	0.00
21	Control	0.00	22.3	8.50	0.00	0.00	0.00	0.21	0.00	0.00
28	PAA	0.04	5.24	8.00	28.8	0.00	0.00	10.5	0.00	0.00
28	PAA	0.00	3.42	8.00	43.7	0.00	0.00	10.5	0.00	0.00
28	PAA	0.00	3.44	8.00	47.3	0.00	0.00	10.2	0.00	0.00
28	PPA	0.11	3.59	8.25	24.8	0.00	0.00	0.00	10.8	0.00
28	PPA	0.00	2.72	8.25	19.6	0.00	0.08	0.00	11.1	0.00
28	PPA	0.22	3.03	8.25	26.5	0.00	0.09	0.00	11.1	0.00
28	PBA	0.00	5.33	8.00	12.2	0.12	0.41	0.00	0.00	9.79
28	PBA	0.00	5.15	8.25	20.0	0.00	0.36	0.00	0.00	10.2
28	PBA	0.00	4.75	8.00	18.5	0.00	0.33	0.00	0.00	9.89
28	PA-mix	0.08	3.59	8.25	25.3	0.00	0.00	3.63	3.56	3.64
28	PA-mix	0.00	4.24	8.25	18.4	0.00	0.21	3.51	3.58	3.65
28	PA-mix	0.04	4.07	8.25	27.7	0.00	0.00	3.24	3.31	3.39
28	Control	0.06	20.6	8.00	0.00	0.00	0.00	0.05	0.00	0.00
28	Control	0.00	17.3	8.00	0.07	0.00	0.00	0.04	0.00	0.00
28	Control	0.14	25.6	8.00	0.14	0.00	0.00	0.05	0.00	0.00

Supplementary Table 3. H_2 and CO_2 as well as VFA (acetate, propionate and butyrate) and PA (PAA, 3-PPA, 3PBA) concentrations, and pH of propionate-fed samples on day 0, 14, 21 and 28

Time [d]	PA-Variant	H_2 [%]	CO_2 [%]	pH	Acetate [mM]	Propionate [mM]	Butyrate [mM]	PAA [mM]	3-PPA [mM]	3-PBA [mM]
0	PAA	0.00	0.00	8.50	0.00	51.6	0.00	10.7	0.00	0.00
0	PAA	0.00	0.00	8.50	0.00	51.0	0.00	10.6	0.00	0.00
0	PAA	0.00	0.00	8.50	0.00	51.9	0.00	10.8	0.00	0.00
0	PPA	0.00	0.00	8.50	0.07	51.0	0.00	0.00	11.1	0.00
0	PPA	0.00	0.00	9.00	0.00	50.5	0.00	0.00	11.2	0.00
0	PPA	0.00	0.00	9.00	0.00	50.8	0.00	0.00	11.2	0.00
0	PBA	0.00	0.00	8.50	0.00	50.8	0.00	0.00	0.00	10.4
0	PBA	0.00	0.00	9.00	0.00	50.9	0.00	0.00	0.00	10.6
0	PBA	0.00	0.00	9.00	0.00	50.6	0.00	0.05	0.00	10.4
0	PA-mix	0.00	0.00	8.50	0.15	49.9	0.00	3.56	3.54	3.80
0	PA-mix	0.00	0.00	9.00	0.00	51.1	0.00	3.64	3.58	3.78
0	PA-mix	0.00	0.00	9.00	0.00	51.2	0.00	3.66	3.61	3.89
0	Control	0.00	0.00	8.00	2.32	51.0	0.00	0.13	0.00	0.00
0	Control	0.00	0.00	8.50	2.47	51.5	0.00	0.13	0.00	0.00
0	Control	0.00	0.00	8.50	2.34	51.0	0.00	0.14	0.00	0.00
14	PAA	0.06	2.50	8.50	1.10	49.0	0.00	10.4	0.00	0.00
14	PAA	0.02	2.12	9.00	1.26	49.7	0.00	10.5	0.00	0.00
14	PAA	0.04	2.26	9.00	1.76	49.5	0.00	10.4	0.00	0.00
14	PPA	0.07	2.49	8.50	1.12	49.4	0.00	0.00	10.8	0.00
14	PPA	0.07	2.33	9.00	0.84	49.2	0.00	0.00	10.9	0.00
14	PPA	0.07	1.78	8.50	1.42	49.1	0.00	0.00	11.1	0.00
14	PBA	0.07	2.46	8.50	1.75	48.2	0.00	0.00	0.00	10.0
14	PBA	0.06	2.12	9.00	1.49	49.3	0.00	0.00	0.00	10.3
14	PBA	0.07	2.13	9.00	1.33	48.0	0.00	0.00	0.00	10.0
14	PA-mix	0.06	2.32	9.00	1.21	47.4	0.00	3.41	3.33	3.54
14	PA-mix	0.05	2.14	9.00	1.35	48.7	0.00	3.52	3.45	3.67
14	PA-mix	0.09	1.93	9.00	1.37	48.7	0.00	3.50	3.45	3.67
14	Control	0.04	11.8	8.00	21.3	16.8	0.00	0.20	0.00	0.00
14	Control	0.03	11.2	8.00	21.2	9.71	0.00	0.07	0.00	0.00
14	Control	0.00	12.1	7.50	23.0	7.95	0.00	0.09	0.00	0.00
21	PAA	0.05	3.50	8.50	1.56	48.0	0.00	10.3	0.00	0.00
21	PAA	0.04	3.17	8.50	2.30	48.7	0.00	10.6	0.00	0.00
21	PAA	0.06	3.15	8.50	2.59	48.2	0.00	10.3	0.00	0.00
21	PPA	0.00	0.00	8.50	1.55	47.3	0.00	0.00	10.5	0.00
21	PPA	0.07	2.28	8.50	2.18	47.4	0.00	0.00	10.7	0.00
21	PPA	0.07	2.75	8.50	2.53	44.4	0.00	0.00	10.4	0.00
21	PBA	0.07	2.99	8.50	4.23	44.4	0.00	0.00	0.00	10.1
21	PBA	0.07	2.79	8.50	3.46	45.0	0.00	0.00	0.00	10.1
21	PBA	0.06	2.69	8.50	3.33	45.1	0.00	0.00	0.00	10.2
21	PA-mix	0.09	2.50	8.50	2.27	47.3	0.00	3.31	3.40	3.66
21	PA-mix	0.06	2.78	8.50	2.24	47.5	0.00	3.48	3.44	3.67
21	PA-mix	0.07	1.93	8.50	2.17	49.2	0.00	3.61	3.55	3.82
21	Control	0.00	11.4	8.00	0.07	0.00	0.00	0.08	0.00	0.00
21	Control	0.00	12.2	8.00	0.00	0.00	0.00	0.10	0.00	0.00
21	Control	0.00	11.1	8.50	0.00	0.00	0.00	0.10	0.00	0.00
28	PAA	0.06	2.66	8.50	3.09	42.4	0.00	9.48	0.00	0.00
28	PAA	0.00	2.61	8.50	6.13	41.8	0.00	10.2	0.00	0.00
28	PAA	0.07	2.25	8.50	5.34	40.2	0.00	9.50	0.00	0.00
28	PPA	0.41	3.06	8.50	3.20	47.8	0.00	0.03	10.9	0.00
28	PPA	0.04	1.91	8.50	6.01	42.4	0.00	0.00	10.7	0.00
28	PPA	0.15	1.91	8.50	7.29	39.1	0.00	0.00	10.5	0.00
28	PBA	0.05	2.35	8.50	18.3	25.5	0.00	0.00	0.00	10.3
28	PBA	0.00	2.72	8.50	13.0	32.2	0.00	0.00	0.00	10.2
28	PBA	0.24	4.32	8.50	13.1	30.9	0.00	0.00	0.00	9.96
28	PA-mix	0.08	2.12	8.50	8.06	37.6	0.00	3.23	3.31	3.54
28	PA-mix	0.00	1.19	8.50	5.60	41.6	0.00	3.40	3.33	3.52
28	PA-mix	0.00	1.85	8.50	5.12	43.7	0.00	3.51	3.45	3.68
28	Control	0.04	19.0	8.00	0.09	0.00	0.00	0.09	0.00	0.00
28	Control	0.04	19.1	8.00	0.00	0.00	0.00	0.09	0.00	0.00
28	Control	0.17	32.9	8.50	0.07	0.00	0.00	0.10	0.00	0.00

Supplementary Table 4. H_2 and CO_2 as well as VFA (acetate, propionate and butyrate) and PA (PAA, 3-PPA, 3PBA) concentrations, and pH of acetate-fed samples on day 0, 14, 21 and 28

Time [d]	PA-Variant	H_2 [%]	CO_2 [%]	pH	Acetate [mM]	Propionate [mM]	Butyrate [mM]	PAA [mM]	3-PPA [mM]	3-PBA [mM]
0	PAA	0.00	0.00	9.00	63.7	0.00	0.00	9.97	0.00	0.00
0	PAA	0.00	0.00	8.50	63.8	0.09	0.00	10.4	0.00	0.00
0	PAA	0.00	0.00	8.50	63.9	0.14	0.00	10.4	0.00	0.00
0	PPA	0.00	0.00	9.00	63.9	0.00	0.00	0.00	11.2	0.00
0	PPA	0.00	0.00	8.50	63.8	0.00	0.00	0.00	11.2	0.00
0	PPA	0.00	0.00	8.50	63.5	0.18	0.00	0.00	11.3	0.00
0	PBA	0.00	0.00	8.50	64.0	0.16	0.00	0.00	0.00	10.2
0	PBA	0.00	0.00	9.00	63.4	0.00	0.00	0.00	0.00	10.1
0	PBA	0.00	0.00	9.00	63.6	0.14	0.00	0.00	0.00	10.1
0	PA-mix	0.00	0.00	9.00	63.9	0.19	0.00	3.48	3.46	3.62
0	PA-mix	0.00	0.00	9.00	63.1	0.33	0.00	3.54	3.46	3.72
0	PA-mix	0.00	0.00	8.50	63.6	0.48	0.00	3.48	3.54	3.69
0	Control	0.00	0.00	8.50	54.3	0.73	0.00	0.13	0.00	0.00
0	Control	0.00	0.00	8.50	60.3	0.71	0.00	0.12	0.00	0.00
0	Control	0.00	0.00	8.50	56.3	0.44	0.00	0.12	0.00	0.00
14	PAA	0.12	1.60	9.00	65.7	0.00	0.00	10.2	0.00	0.00
14	PAA	0.15	1.77	9.00	65.1	0.00	0.00	10.2	0.00	0.00
14	PAA	0.05	1.50	8.50	62.3	0.00	0.00	9.77	0.00	0.00
14	PPA	0.05	1.38	8.50	63.0	0.00	0.00	0.00	10.7	0.00
14	PPA	0.05	1.94	8.50	63.5	0.00	0.00	0.00	10.9	0.00
14	PPA	0.04	2.06	9.00	63.7	0.00	0.00	0.00	10.9	0.00
14	PBA	0.04	1.74	9.00	64.8	0.00	0.00	0.00	0.00	10.0
14	PBA	0.03	1.71	8.50	64.7	0.00	0.00	0.00	0.00	10.1
14	PBA	0.05	1.82	9.00	65.7	0.00	0.00	0.00	0.00	10.2
14	PA-mix	0.04	1.55	8.50	66.5	0.00	0.00	3.45	3.54	3.69
14	PA-mix	0.22	1.88	9.00	64.4	0.00	0.00	3.52	3.46	3.77
14	PA-mix	0.05	1.84	9.00	65.0	0.00	0.00	3.40	3.44	3.33
14	Control	0.00	7.47	8.50	0.00	0.00	0.00	0.00	0.00	0.00
14	Control	0.00	7.32	8.50	1.75	0.52	0.00	0.17	0.00	0.00
14	Control	0.00	6.82	8.50	3.14	0.50	0.00	0.17	0.00	0.00
21	PAA	0.04	1.75	8.50	62.5	0.00	0.00	10.4	0.00	0.00
21	PAA	0.04	1.61	8.50	62.7	0.00	0.00	10.3	0.00	0.00
21	PAA	0.06	1.54	8.50	62.7	0.00	0.00	10.3	0.00	0.00
21	PPA	0.08	1.82	8.50	63.2	0.00	0.00	0.00	11.0	0.00
21	PPA	0.04	1.76	8.50	63.6	0.00	0.00	0.00	11.1	0.00
21	PPA	0.04	1.82	8.50	59.7	0.00	0.00	0.00	11.1	0.00
21	PBA	0.04	1.75	8.50	0.09	0.00	0.00	0.00	0.00	10.2
21	PBA	0.05	1.76	8.50	58.1	0.00	0.00	0.00	0.00	10.1
21	PBA	0.05	1.83	8.50	58.5	0.00	0.00	0.00	0.00	10.2
21	PA-mix	0.04	1.65	8.50	62.1	0.00	0.00	3.59	3.49	3.80
21	PA-mix	0.03	1.79	8.50	57.8	0.00	0.00	3.56	3.49	3.81
21	PA-mix	0.04	1.76	8.50	59.3	0.00	0.00	3.52	3.42	3.70
21	Control	0.00	13.5	8.50	0.79	0.00	0.00	0.19	0.00	0.00
21	Control	0.08	10.6	8.50	0.86	0.22	0.00	0.20	0.00	0.00
21	Control	0.00	12.6	8.50	0.83	0.75	0.00	0.19	0.00	0.00
28	PAA	0.02	2.54	8.50	45.6	0.00	0.00	10.4	0.00	0.00
28	PAA	0.00	1.96	8.50	48.8	0.00	0.00	10.1	0.00	0.00
28	PAA	0.05	1.30	8.50	51.5	0.00	0.00	10.2	0.00	0.00
28	PPA	0.06	2.07	8.50	57.3	0.00	0.00	0.00	11.0	0.00
28	PPA	0.00	1.92	8.50	55.6	0.00	0.00	0.00	11.2	0.00
28	PPA	0.00	1.91	8.50	35.0	0.00	0.00	0.00	11.0	0.00
28	PBA	0.00	2.27	8.50	36.2	0.00	0.00	0.00	0.00	10.1
28	PBA	0.00	2.10	8.50	35.0	0.00	0.00	0.00	0.00	10.2
28	PBA	0.03	1.64	8.50	37.1	0.00	0.00	0.00	0.00	10.1
28	PA-mix	0.00	2.05	8.50	47.4	0.00	0.00	3.51	3.46	3.75
28	PA-mix	0.04	1.65	8.50	35.0	0.00	0.00	3.37	3.45	3.74
28	PA-mix	0.04	1.74	8.50	38.9	0.00	0.00	3.55	3.45	3.71
28	Control	0.03	13.8	8.50	0.00	0.00	0.00	0.19	0.00	0.00
28	Control	0.00	14.6	8.50	0.37	0.00	0.00	0.20	0.00	0.00
28	Control	0.00	13.4	8.50	0.55	0.00	0.00	0.21	0.00	0.00

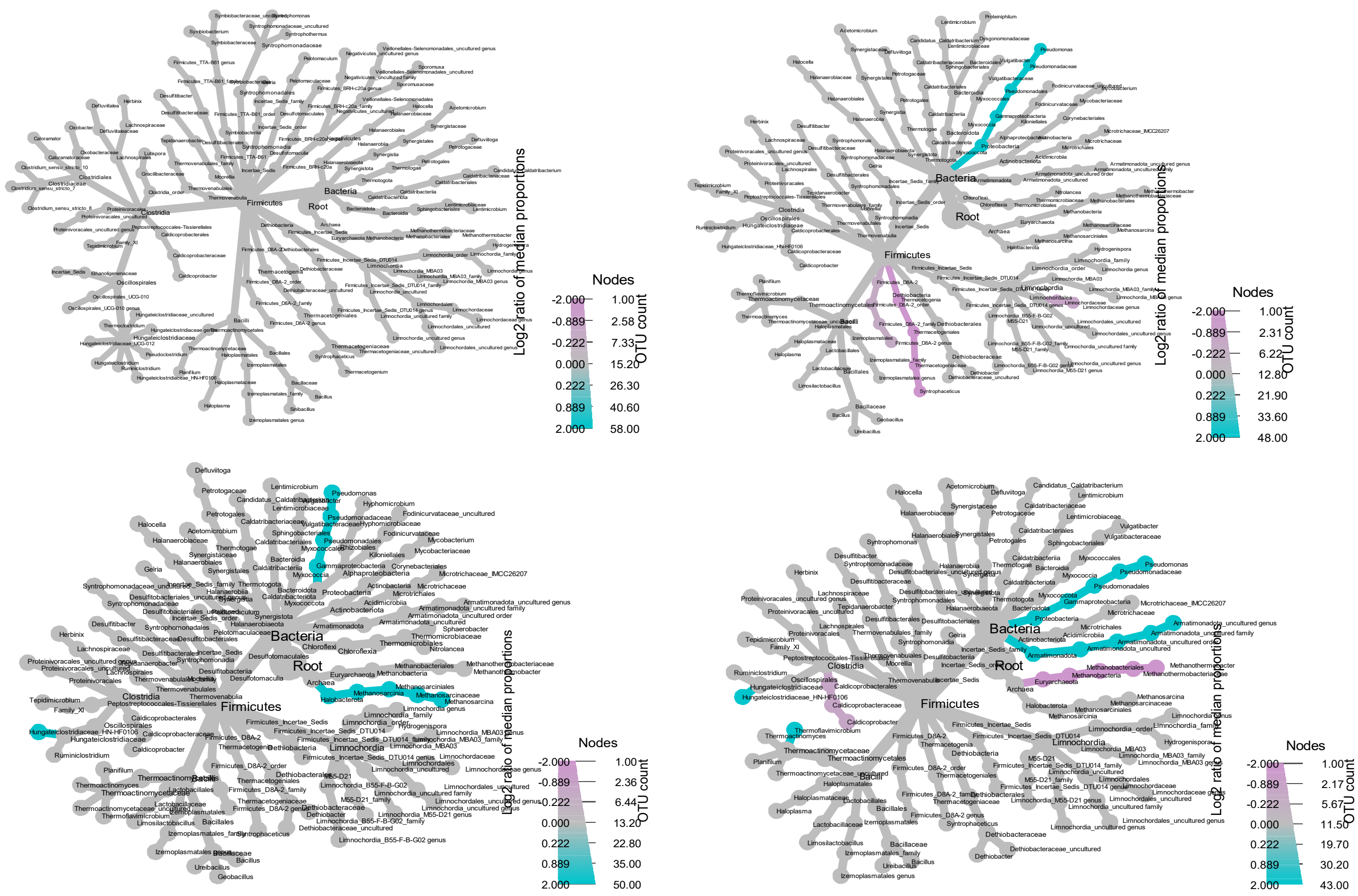

Supplementary Figure 1. Heat trees for MCC, acetate, propionate and butyrate reactors showing significant taxa for PA addition (PAA, PPA, PBA, PA-mix reactors, pink) and for controls (no PA addition, turquoise).

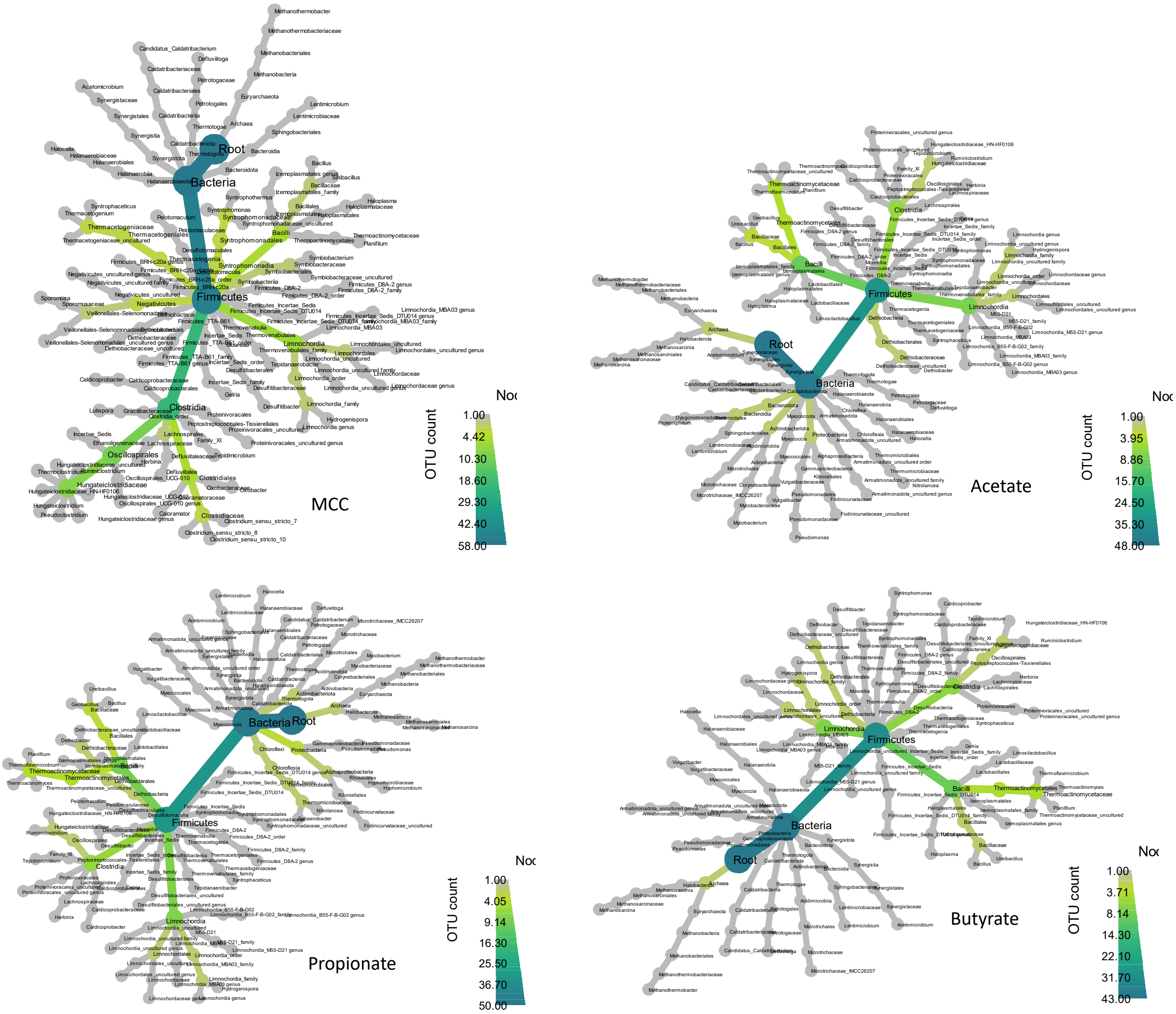

Supplementary Figure 2. Heat trees showing OTU counts (microbial diversity) for MCC (top, left), acetate (top, right), propionate (bottom, left) and butyrate reactors (bottom, right) over all PA variations.

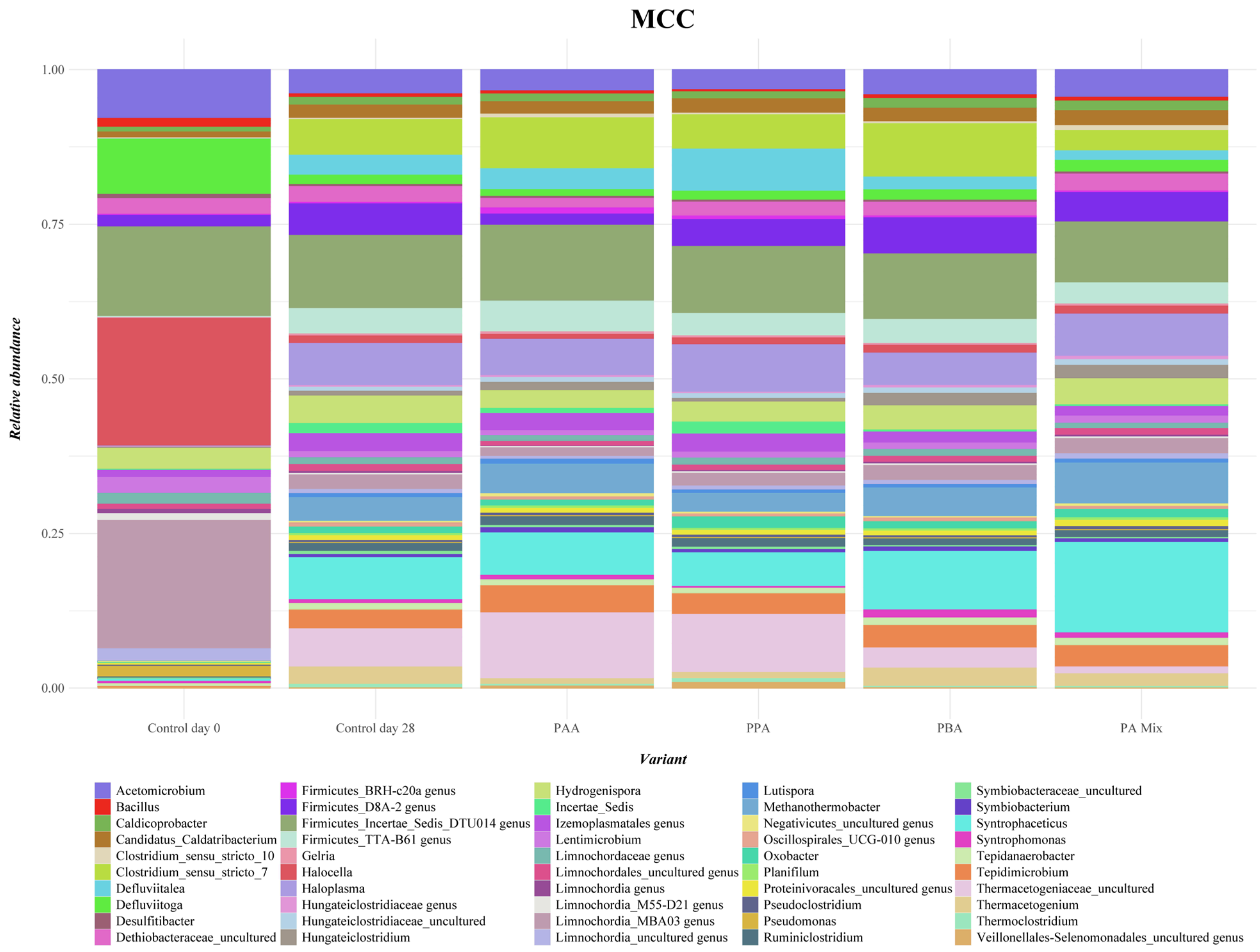

Supplementary Figure 3. Relative microbial abundances of the 50 most abundant genera of MCC reactors exposed to 0 mM PA, or to 10 mM PAA, PPA, PBA or PA-mix after 28 days. Controls on day 0 are also included to show the initial microbial composition of the inoculum.

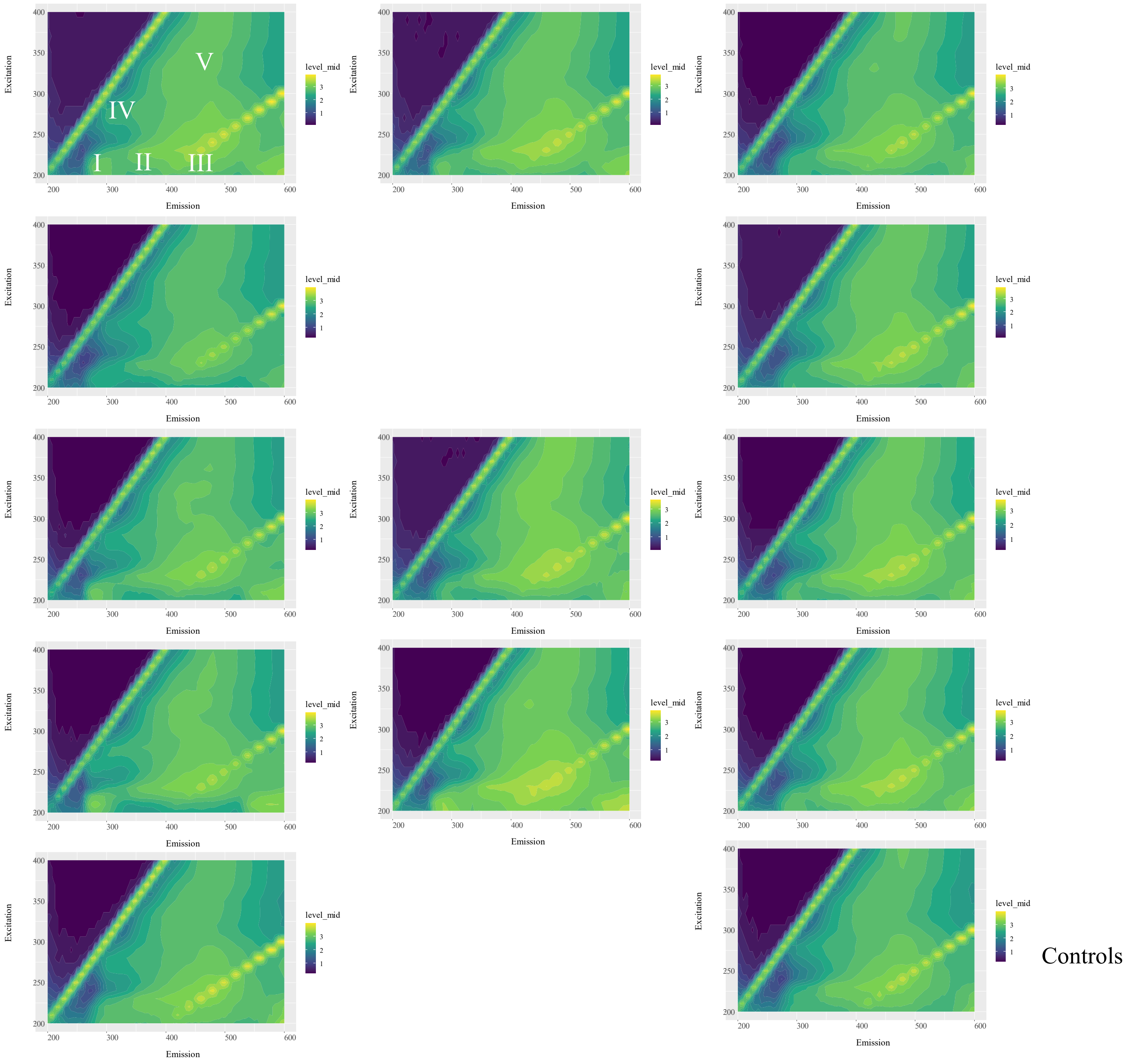

Supplementary Figure 4. Excitation-emission matrices of <u>MCC</u> reactors for each PA variation and parallel (P1 – P3). Fluorescence data were $log_{10}(x+1)$ transformed and range from 0 (dark violet) to 3 (yellow). I – V show areas typical for specific EPS types – this caption counts for all matrices.

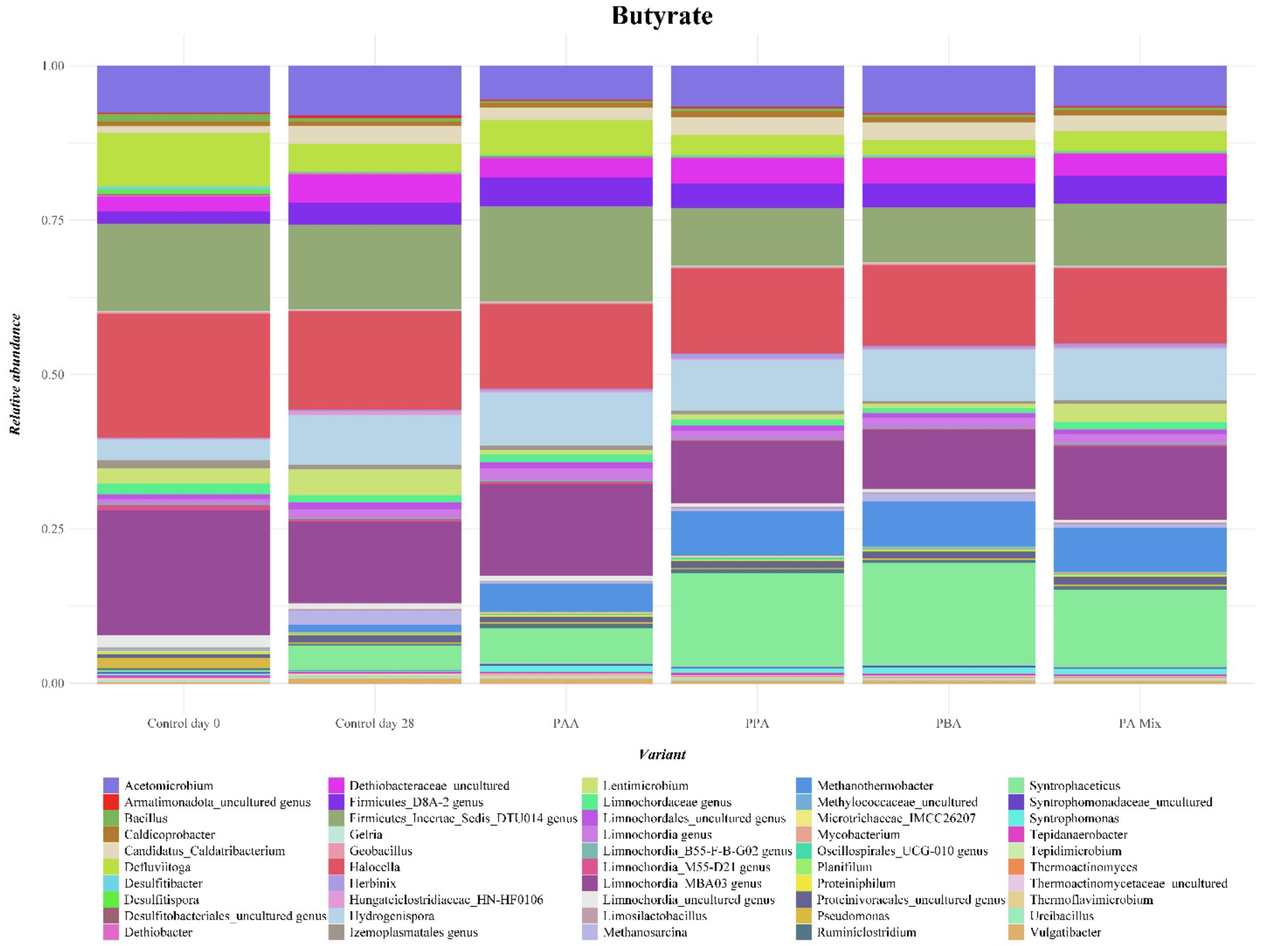

Supplementary Figure 5. Relative microbial abundances of the 50 most abundant genera of <u>butyrate reactors</u> exposed to 0 mM PA, or to 10 mM PAA, PPA, PBA or PA-mix after 28 days. Controls on day 0 are also included to show the initial microbial composition of the inoculum.

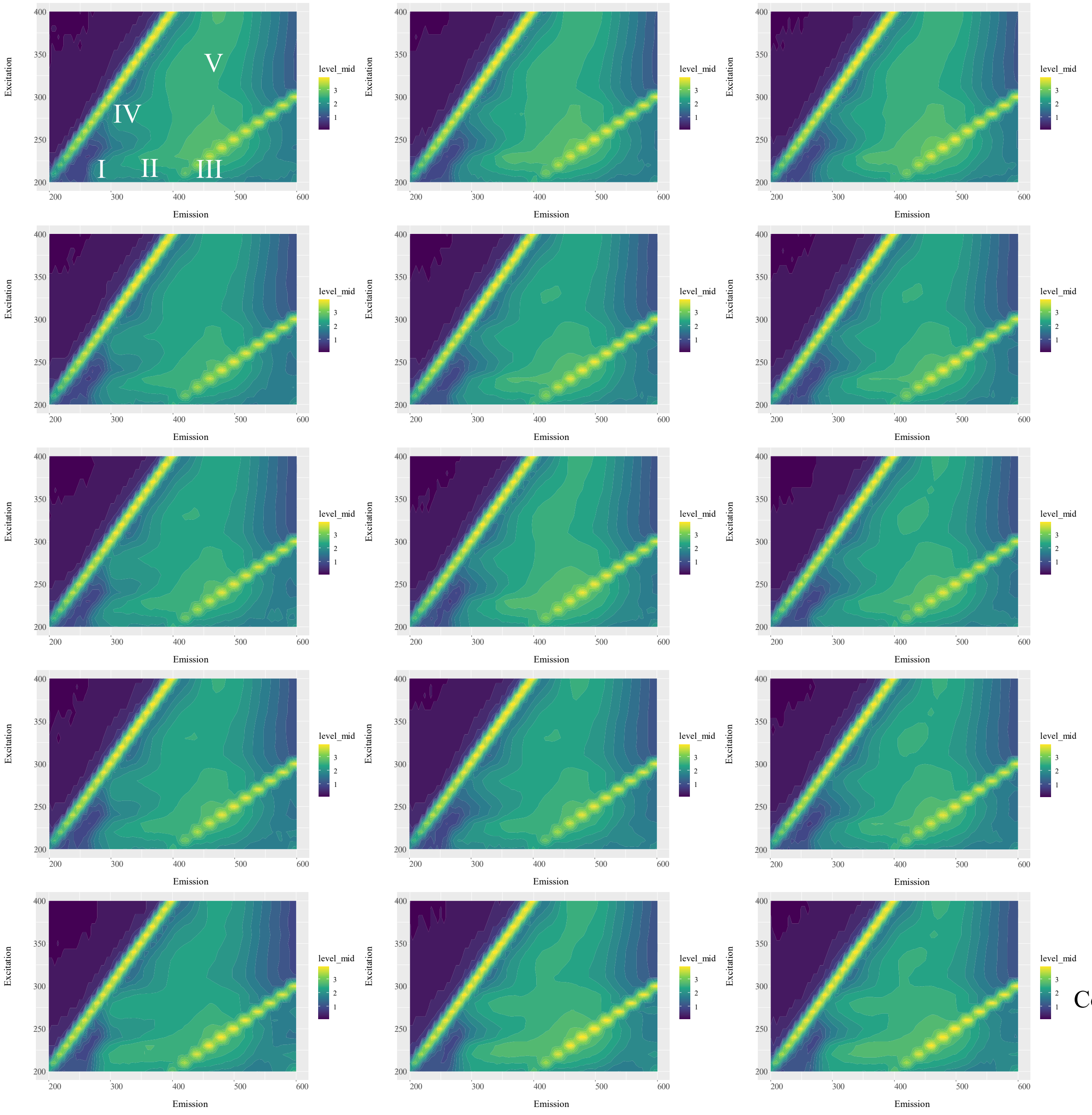

Controls

Supplementary Figure 6. Excitation-emission matrices of butyrate reactors for each PA variation and parallel (P1 – P3). Fluorescence data were $\log_{10}(x+1)$ transformed and range from 0 (dark violet) to 3 (yellow). I – V show areas typical for specific EPS types – this caption counts for all matrices.

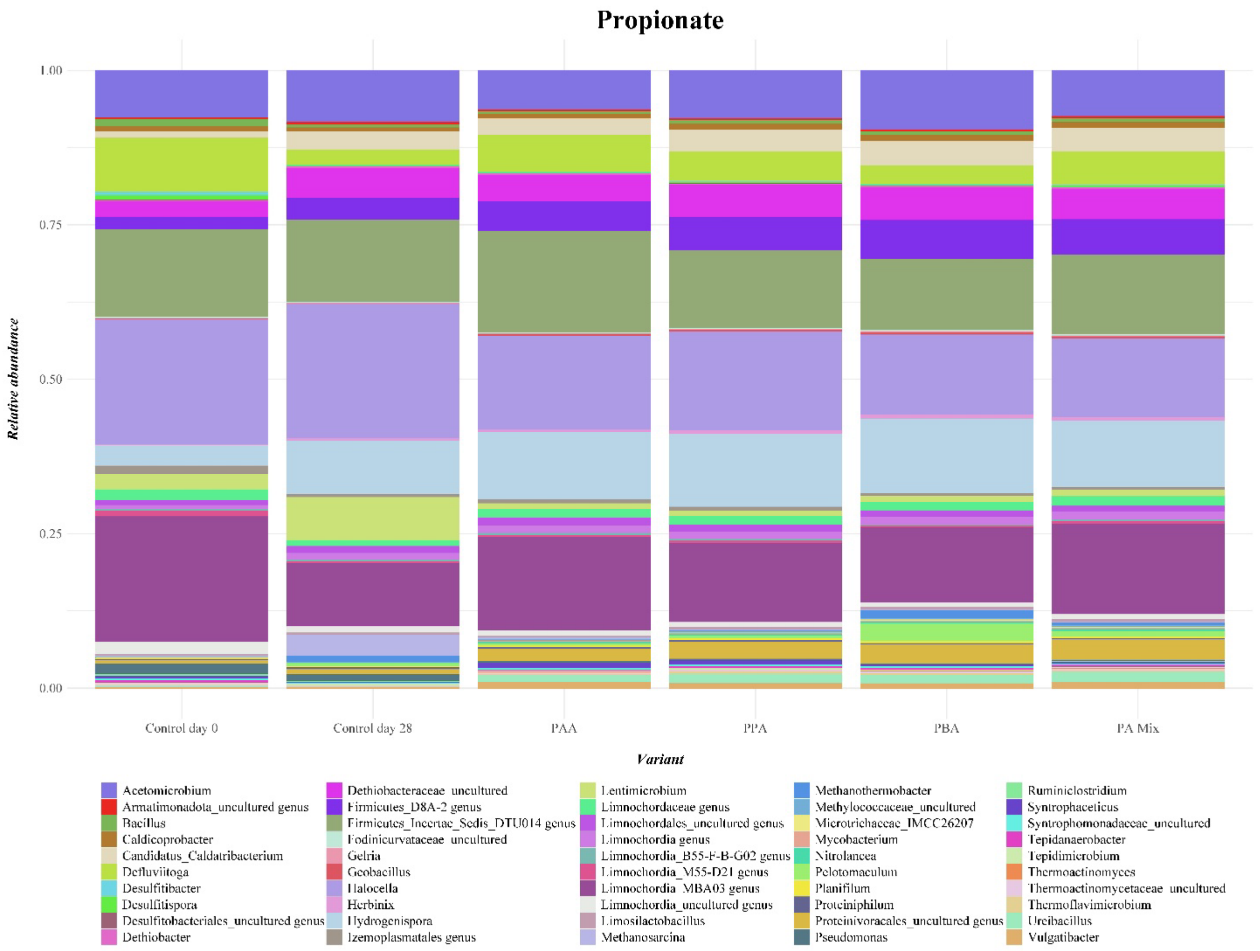

Supplementary Figure 7. Relative microbial abundances of the 50 most abundant genera of <u>propionate reactors</u> exposed to 0 mM PA, or to 10 mM PAA, PPA, PBA or PA-mix after 28 days. Controls on day 0 are also included to show the initial microbial composition of the inoculum.

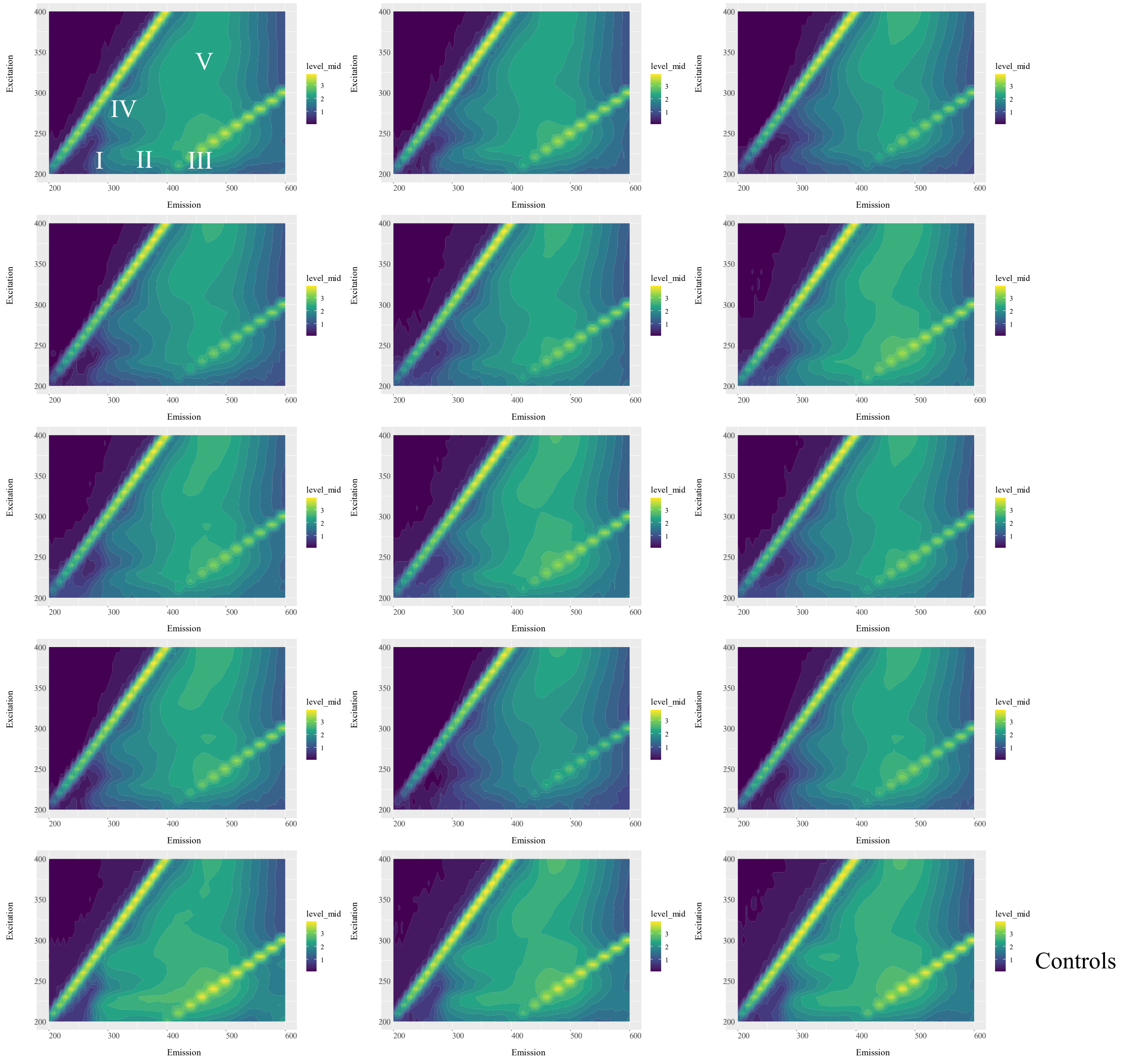

Supplementary Figure 8. Excitation-emission matrices of <u>propionate</u> reactors for each PA variation and parallel (P1 – P3). Fluorescence data were $\log_{10}(x+1)$ transformed and range from 0 (dark violet) to 3 (yellow). I – V show areas typical for specific EPS types – this caption counts for all matrices.

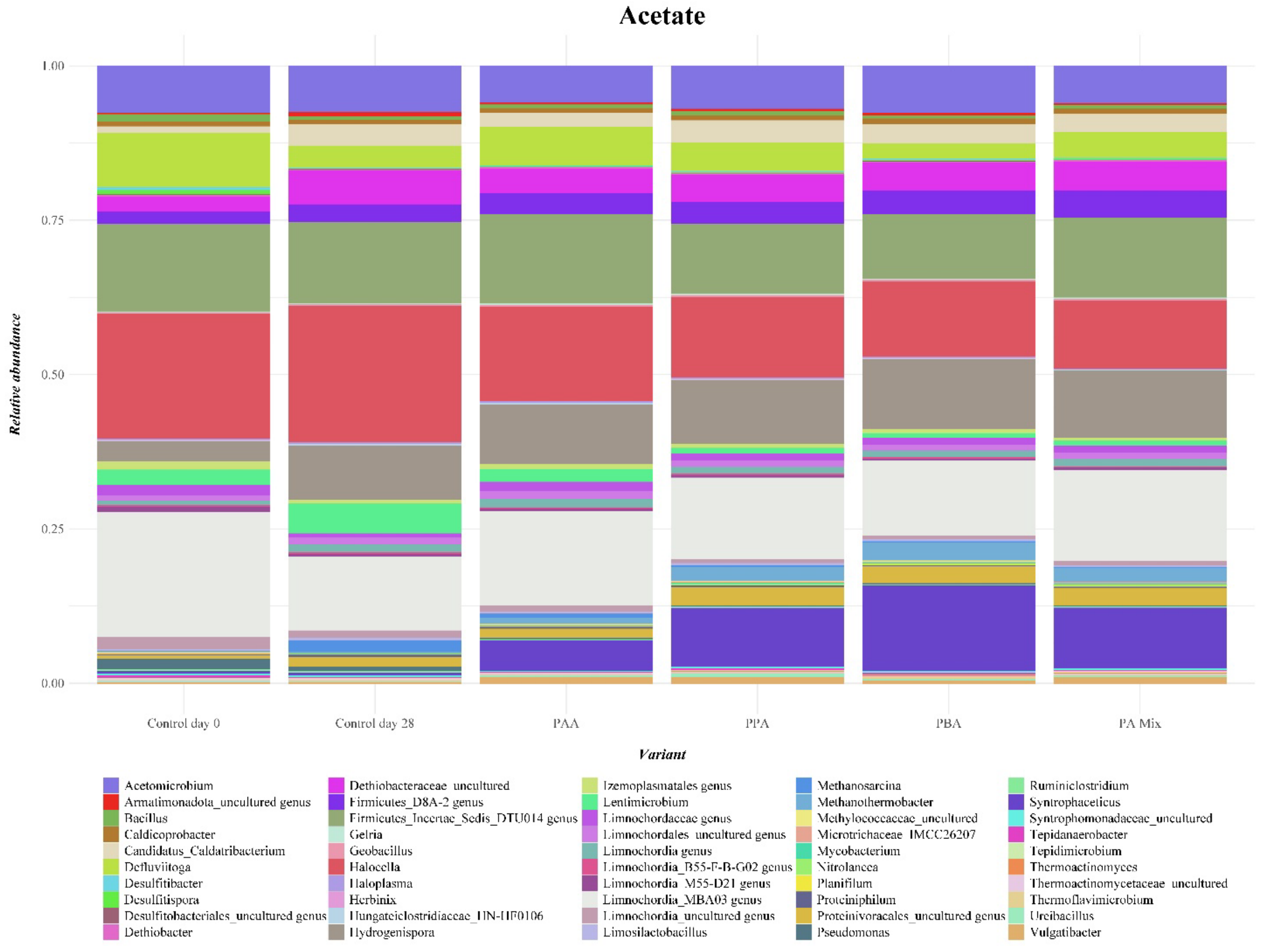

Supplementary Figure 9. Relative microbial abundances of the 50 most abundant genera of acetate reactors exposed to 0 mM PA, or to 10 mM PAA, PPA, PBA or PA-mix after 28 days. Controls on day 0 are also included to show the initial microbial composition of the inoculum.

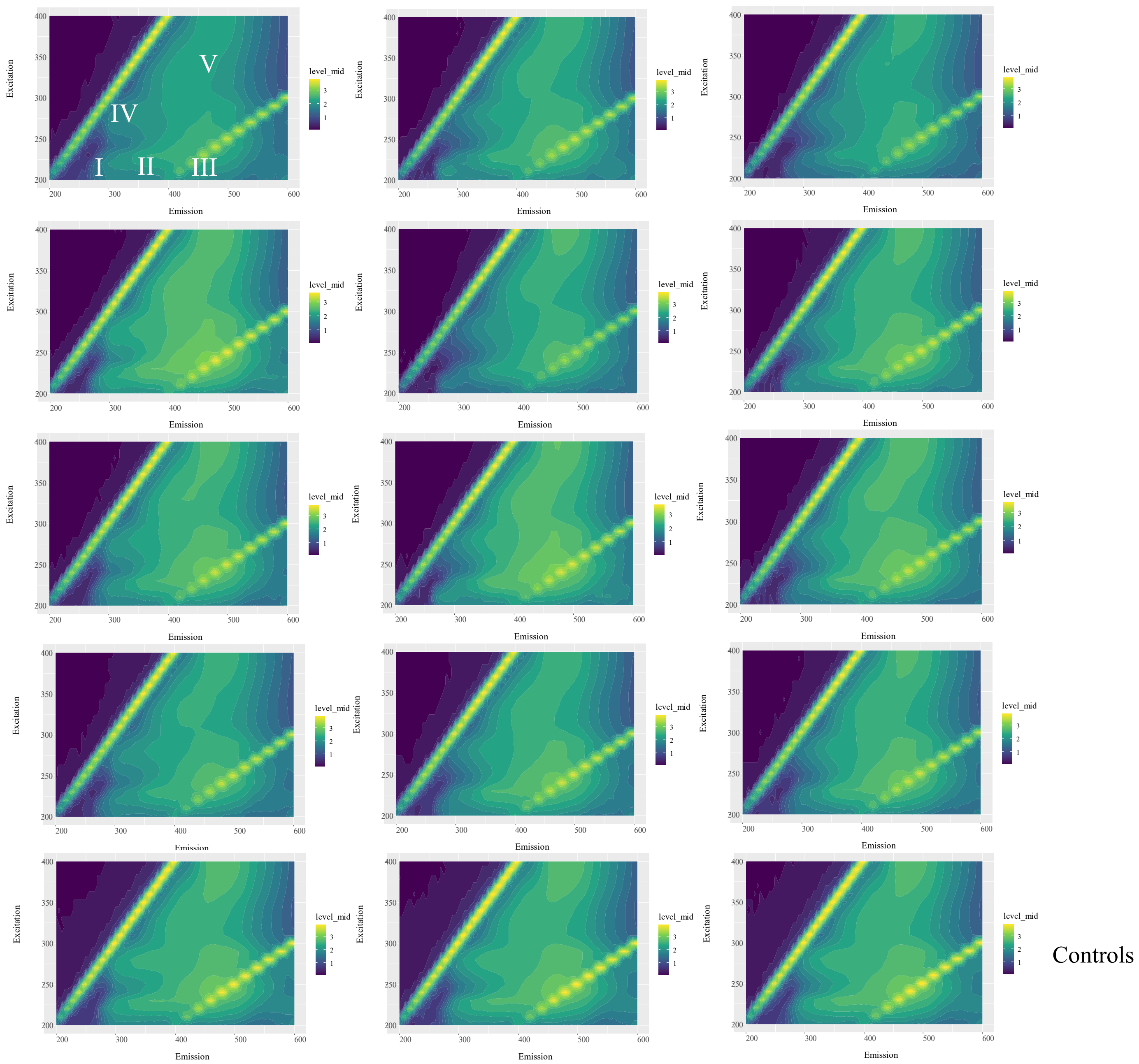

Supplementary Figure 10. Excitation-emission matrices of <u>acetate</u> reactors for each PA variation and parallel (P1 – P3). Fluorescence data were $\log_{10}(x+1)$ transformed and range from 0 (dark violet) to 3 (yellow). I – V show areas typical for specific EPS types – this caption counts for all matrices.
